# Automated Digital Interventions and Smoking Cessation: Systematic Review and Meta-analysis Relating Efficiency to a Psychological Theory of Intervention Perspective

**DOI:** 10.2196/38206

**Published:** 2022-11-16

**Authors:** Leihao Sha, Xia Yang, Renhao Deng, Wen Wang, YuJie Tao, HaiLing Cao, Qianshu Ma, Hao Wang, Yirou Nie, Siqi Leng, Qiuyue Lv, Xiaojing Li, Huiyao Wang, Yajing Meng, Jiajun Xu, Andrew J Greenshaw, Tao Li, Wan-jun Guo

**Affiliations:** 1 Mental Health Center and Sichuan Clinical Medical Research Center for Mental Disorders West China Hospital of Sichuan University Chengdu China; 2 Chinese Evidence-Based Medicine Center West China Hospital of Sichuan University Chengdu China; 3 Department of Psychiatry University of Alberta Edmonton, AB Canada; 4 Mental Health Center and Hangzhou Seventh People's Hospital Zhejiang University School of Medicine Hangzhou China

**Keywords:** smoking cessation, automated, digital intervention, psychological theory, meta-analysis, systematic review, public health, side effects, interventions, randomized controlled trial, self-help

## Abstract

**Background:**

Smoking remains a highly significant preventable global public health problem. In this context, digital interventions offer great advantages in terms of a lack of biological side effects, possibility of automatic delivery, and consequent human resource savings relative to traditional interventions. Such interventions have been studied in randomized controlled trials (RCTs) but have not been systematically reviewed with the inclusion of text-based and multiplatform-based interventions. In addition, this area has not been evaluated from the perspective of the psychological theoretical basis of intervention.

**Objective:**

The aim of this paper is to assess the efficiency of digital interventions in RCT studies of smoking cessation and to evaluate the effectiveness of the strategies used for digital interventions.

**Methods:**

An electronic search of RCTs was conducted using PubMed, Embase, and the Cochrane Library by June 30, 2021. Eligible studies had to compare automated digital intervention (ADI) to the use of a self-help guideline or no intervention. Participants were current smokers (aged 16 years or older). As the main outcome, abstinence after endpoint was extracted from the studies. Systematic review and meta-analysis were conducted to assess the efficiency of ADIs. Metaregressions were conducted to assess the relationship between intervention theory and effectiveness.

**Results:**

A total of 19 trials (15,472 participants) were included in the analysis. The overall abstinence rate (95% CI) at the endpoint was 17.8% (17.0-18.7). The overall risk ratio of the intervention group compared to the controls at the endpoint was 17.8% (17.0-18.7). Cochrane risk-of-bias tool for randomized trials (ROB 2) suggested that most of the studies had a low risk of bias (56.3%). Psychological theory–related constructs or predictors, which refer to other theory-based concepts (rather than only behavioral theory) such as craving or anxiety, are associated with effectiveness.

**Conclusions:**

This study found that ADI had a clear positive effect compared to self-help guidelines or to no intervention, and effectiveness was associated with theory-related constructs or predictors. ADIs should be promoted by policy makers and clinical practitioners to address the huge gap between the need for smoking cessation and availability of traditional treatment resources. Possible increases in ADI efficiency may be achieved by optimally integrating psychotherapeutic theories and techniques.

**Trial Registration:**

PROSPERO CRD42021256593; https://www.crd.york.ac.uk/prospero/display_record.php?RecordID=256593

## Introduction

### Background

Smoking tobacco is the leading risk factor for noncommunicable diseases and the leading cause of substance-attributable mortality rates and disability-adjusted life years [1,2].

Most smokers use tobacco products constantly or relapse after quitting due to nicotine addiction. Therapies for smoking cessation include pharmacological agents and psychosocial approaches [3]. Pharmacotherapy is recommended for short-term use [4]. Food and Drug Administration–approved first-line medications include nicotine replacement therapy (NRT), bupropion, and varenicline [5], which generally result in higher quit rates than placebo [6]. Side effects of these medications, including nausea, vomiting, and neuropsychiatric symptoms, often limit use of such medication treatment for many general smokers [7,8]. Counseling and behavioral therapies are also effective in smoking cessation [9,10]. Compared to pharmacotherapy, counseling and behavioral therapies may enhance patients’ motivation and provide education on general and specific strategies for smoking cessation and encouragement [5]. The shortage of trained counselors remains a barrier not only for general availability but also effectiveness of nonmedication interventions. Current inadequacies in availability of professional human resources, inequities in primary care, overwhelming serious cases in hospitals, unsatisfactory accessibility, poor cost-effectiveness issues, and lack of compliance restrict the efficiency of smoking cessation interventions and result in numerous untreated or relapsed smokers [3,11]. Such evidence indicates a pressing need for more cost-effective interventions.

With the progress of mobile and digital technologies, mobile health management and digital therapeutics present good prospects for managing chronic health conditions [12-14], especially in low- and middle-income countries (LMIC) [15]; this is because they have the advantages of having fewer side effects than pharmacotherapy, less need for trained human resources than counseling and behavioral therapies because of the possibility of automatic delivery, more individualized interventions, as well as great accessibility and portability, which may result in higher cost-effectiveness [11,12,16,17]. Such technologies are, therefore, potentially effective strategies for improving health delivery, especially in LMIC, where the lack of capacity in professional human and related resources is prominent [10]. Recent studies report positive indications that automated digital interventions (ADIs) are well accepted and may benefit smokers in LMICs [13,14], although the number of studies focusing on effectiveness was fewer than those investigating acceptability and feasibility of ADIs in LMICs [15,18,19]. For intervention content, earlier digital interventions provided text interventions (text-based), while multiplatform-based interventions, which provided diverse tools for interventions such as serious games and virtual reality, have been developed and studied more recently. Evidence indicates that mobile health management and digital interventions could be effective in many chronic conditions, including hypertension [20,21], diabetes [22,23], and mental illness [24,25]. Digital interventions may also be promising treatments for substance use disorders other than tobacco smoking, in relation to narrowing the enormous gap between the growing need and lack of professional human resources [26-28]. More than 30 clinical trials examining effectiveness of digital smoking cessation interventions have been conducted. A systemic review focused on text-based interventions has documented promising results [29] but did not provide analysis for ADIs. Neither systematic reviews nor meta-analyses are available that evaluate text-based and multiplatform-based interventions, although the latter are anticipated to be more acceptable to patients.

### Theoretical Basis of Interventions

Effectiveness of digital interventions could be influenced by many aspects including psychological behavioral theories, which are often used to develop content of messages used for a respective intervention [30], environment [31], and strategies of implementation [32]. Many retrospective studies found that applying theories more appropriately could improve effectiveness through the implementation of theory-based interventions [33-35]. Interventions guided by different theories could result in different effectiveness. For example, regarding health-related behavior, it is reported that interventions based on the theory of planned behavior tended to have substantially greater effects than other theories such as the transtheoretical model or the elaboration likelihood model [36]. The theory coding scheme (TCS) is a tool for describing the theoretical basis of interventions [37] and is widely used in meta-analyses [31,36,38] for evaluating theory use. The TCS has different items and categories describing relevant theoretical constructs. The importance of the association between theoretical basis and intervention effectiveness for smoking cessation has not yet been assessed, although some reviews that focused on other disorders or conditions demonstrated diverse evidence for the relationship of intervention effectiveness to intervention theory with both positive [36,38] and null [30,39] associations.

### Objective

To enhance smoking cessation interventions with ADIs, this systemic review plus meta-analysis aimed to assess the efficiency of automated digital interventions in randomized controlled trial (RCT) studies and to evaluate the association between intervention effectiveness and how the intervention strategy is based on theory.

## Methods

### Data Sources and Search

Through a broad search of databases, including PubMed, Embase, CNKI, and Cochrane Library, we identified relevant studies using the PRISMA (Preferred Reporting Items for Systematic Reviews and Meta-Analyses) guidelines [40]. We also checked the reference lists of the included studies and relevant reviews for study selection. The search strategy combined terms related to digital intervention (ie, mobile health) and tobacco smoking cessation (ie, nicotine addiction). For the full search strategy, see Supporting Information 1 in Multimedia Appendix 1.

### Inclusion and Exclusion Criteria and Quality Assessment

We included studies with the following criteria: (1) RCT; (2) study with participants aged >16 years and current tobacco smokers; (3) intervention automatedly delivered via a digital method and targeted smoking cessation; (4) abstinence assessment during the whole follow-up period of at least 3 months; (5) self-help guidelines or no intervention in control group; and (6) studies reported in English or Chinese. To focus on smoking cessation solely, study participants with other mental diseases are excluded from this analysis. Moreover, interventions that include financial incentives (which could limit the generalization of ADIs) and studies with rate of loss to follow-up over 60% are excluded from the analysis. No exclusion was made for duration of intervention, intervention frequency, study region study sample, or the content of the delivery or frequency of the messages.

Two authors independently reviewed the studies using the inclusion and exclusion criteria. The title and abstract of each study were screened initially, followed by full-text analysis if the title and abstract were consistent with the inclusion and exclusion criteria. Disagreement on evaluation for inclusion was resolved by discussion of the authors, and if necessary, a third reviewer was included in the discussion to reach consensus.

For quality assessment, we used the Cochrane risk of bias tool (Cochrane ROB 2) [41], which is a commonly used tool to access bias for clinical trials. The randomization process, deviations from intended interventions, missing outcome data, measurement of the outcome, and selection of the reported result were assessed to obtain an estimate of overall bias.

### Data Extraction and Effect Size Calculation

We extracted follow-up data at the endpoint (abstinence during the whole follow-up period) as the main outcome for efficiency assessment and follow-up data at 3 months and 6 months (if available) as secondary outcomes. We also extracted author, publication year, study region, sample size, control type, intervention type, and intervention duration of all included articles as baseline information (Table 1). As key components of digital intervention, the timing was consistent across studies; all studies began their intervention around the quit date. Frequency was not reported in most of the studies as their intervention could be available whenever the participant wants or on a daily basis because of automated delivery. Therefore, we did not conduct further analyses in these aspects. We used the risk ratio (RR) as the efficiency measure, and RRs were calculated with follow-up data extracted from the included articles. When cases were reported as lost to follow-up, they were treated as relapse.

We used the TCS to assess the potential relationship between intervention theory and intervention effectiveness [42]. The TCS evaluates several aspects of a theory-based intervention, and these aspects will be analyzed, including “is theory mentioned?” “are the relevant theoretical constructs targeted?” “is theory used to select recipients or tailor interventions?” “are the relevant theoretical constructs measured?” “is theory tested?” and “is theory refined.” Two independent reviewers coded all included articles. In the case of differing opinions, consensus was achieved by discussion. The amended version of TCS was used in this analysis. Two items (“quality of measures” and “randomization of participants to condition”) were excluded from the amended vision of the TCS because these aspects were assessed in Cochrane ROB 2 previously [41]. The amended TCS has a total of 22 items, including all subitems. Each item was coded as 1 (present) or 0 (absent). All intervention theories mentioned in the study or in the reference list were recognized during the review process. The TCS also had 6 categories of theory use, and total scores of each category were calculated for further analysis.

### Data Analysis

We conducted all analyses in R (version 4.0.4; R Foundation for Statistical Computing). Q statistic and I^2^ were reported for study heterogeneity. If the I^2^ was at least 40% and the Q statistic was significant (*P*<.05), the overall effect was considered heterogeneous [37]. We used a random-effects model to analyze the overall effect. We used intention-to-treat analysis to assess all data, which handles those cases lost to follow-up as relapse. To test the accuracy of the overall effects, sensitivity analysis was performed. We also conducted subgroup analysis for 3-month and endpoint abstinence to test the source of heterogeneity using baseline information. Funnel plot and Peters tests were employed to test the publication bias of this analysis because of the sample size [43]. Effect sizes of all studies with 95% CI and the weighted aggregate effects were represented using forest plots. The abstinence rates during follow-up (3 months, 6 months, and endpoint) were calculated and reported with a 95% CI for individual studies and overall.

To evaluate the association between intervention effectiveness and how the intervention strategy is based on theory, we conducted univariate and multivariate metaregressions for 3-month, 6-month, and endpoint abstinence with the TCS score (including each item, each category, and total score). If none of the studies or all of the studies met the standards of the item, no metaregression was conducted for this item. All items or categories that showed a significant association with effectiveness and were coded by more than one study were included in the multivariate regression analysis. The regression coefficient (B) represented the mean of the unstandardized effects that differentially included each TCS covariate. The regression coefficients were calculated and reported with 95% CI and *P* values.

## Results

### Study Characteristics

**Figure 1:**
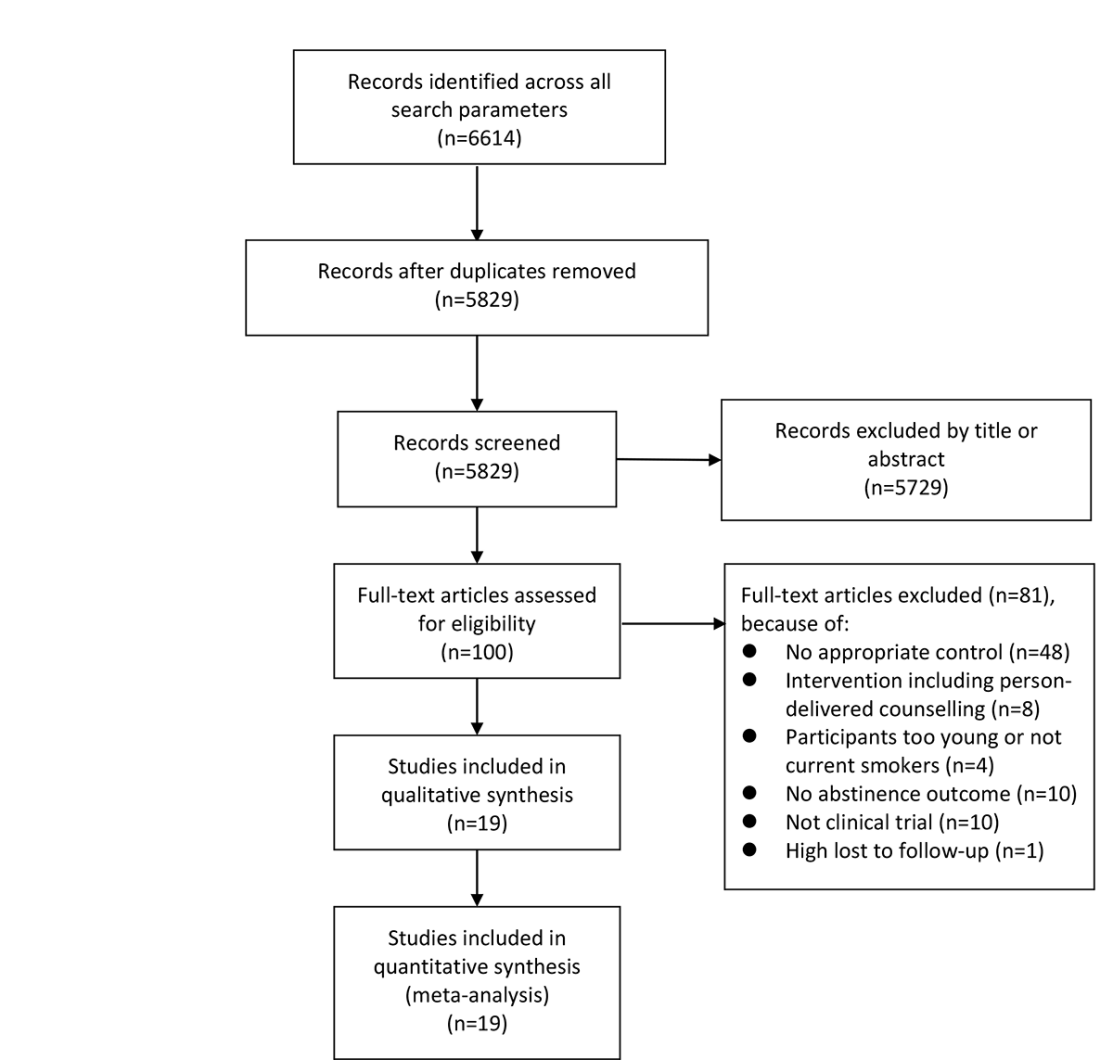
Flowchart.

A total of 6614 studies were identified after initial database search, and a total of 5829 studies were retained after removing duplicates. After reviewing the full texts, 19 studies were included in the analysis. For the details of study selection, see Figure 1. Table 1 demonstrates basic characteristics for each ADI clinical trial. Out of the 19 trials, 6 (32%) performed no intervention in the control group, and the interventions in the other 13 (68%) trials comprised use of self-help guidelines. Moreover, 8 (42%) trials tested effectiveness for text-based intervention, and the other 11 (58%) trials tested multiplatform-based interventions. Of all the reported theory uses, the transtheoretical model of behavior change (13/19, 68.42%) and cognitive behavioral therapy (12/19, 63.16%) were the most commonly used. The endpoints of included studies ranged from 3 months to 12 months. The sample size varied from 110 to 2478, with 9 trials over 500. Overall abstinence rates (3 months, 6 months, and endpoint) were demonstrated with 95% CIs in Table 1. The results from the Peters test and funnel plots suggested a low risk of publication bias with *P*>.05 (for details, see Supporting Information 2 in Multimedia Appendix 1). Total between-study heterogeneity was significant for 3-month abstinence, 6-month abstinence, and endpoint abstinence (3-month: *X*^2^=74.04, *P*<.05 and *I*^2^=76%; 6-month: *X*^2^=63.40, *P*<.05 and *I*^2^=81%; endpoint: *X*^2^=90.10, *P*<.05 and *I*^2^=80% endpoint). With respect to quality assessment, Cochrane ROB 2 suggested that, of the 19 included studies, 11 (58%) had a low risk of bias, 3 (16%) showed some concern of bias, and 5 (26%) showed a high risk of bias (Supporting Information 3 in Multimedia Appendix 1).

**Table 1:** Descriptive information of included clinical trials.

Author^a^	Year	Study region	Intervention type	Control type	Endpoint	Sample size, n (%)	Abstinence, percentage^b^ (95% CI)				
							3-month			Endpoint	
							Experiment	Control	Experiment		Control
Abroms [44]	2014	United States	Text-based	Self-help guideline	6 months	503 (3.25)	27.5 (22.2, 33.3)	16.2 (11.8, 21.5)	11.1 (7.5, 15.5)		5.0 (2.6, 8.5)
Baskerville [45]	2018	Canada	Multiplatform	Self-help guideline	6 months	1599 (10.33)	8.8 (6.9, 10.9)	9.1 (7.2, 11.4)	6.1 (4.6, 8.0)		1.5 (1.0, 2.7)
BinDhim [46]	2018	United States, etc^c^	Multiplatform	Self-help guideline	6 months	684 (4.42)	17.3 (13.4, 21.7)	7.9 (5.3, 11.3)	10.2 (7.2, 13.9)		4.7 (2.7, 7.5)
Brendryen [47]	2008	Norway	Multiplatform	Self-help guideline	12 months	290 (1.87)	30.0 (22.5, 38.0)	11.6 (6.9, 18.0)	20.1 (13.9, 27.6)		6.8 (3.3, 12.2)
Kraft [48]	2008	Norway	Multiplatform	Self-help guideline	12 months	396 (2.56)	44.7 (37.6, 51.9)	28.6 (22.5, 35.5)	37.6 (30.8, 44.7)		24.1 (18.4, 30.7)
Goldenhersch [49]	2020	Argentina	Multiplatform	Self-help guideline	3 months	120 (0.78)	33.3 (21.7, 46.7)	5.0 (1.0, 13.9)	33.3 (21.7, 46.7)		5.0 (1.0, 13.9)
Mavrot [50]	2017	Switzerland	Text-based	Self-help guideline	6 months	1120 (7.24)	20.2 (17.0, 23.8)	17.5 (14.4, 20.9)	17.0 (14.0, 20.3)		15.5 (12.6, 18.8)
Scholten [51]	2019	Netherlands	Multiplatform	Self-help guideline	3 months	144 (0.93)	29.2 (19.0, 41.1)	27.8 (17.9, 39.6)	29.2 (19.0, 41.1)		27.8 (17.9, 39.6)
Whittaker [52]	2011	New Zealand	Multiplatform	Self-help guideline	6 months	226 (1.46)	27.3 (19.2, 36.6)	21.6 (14.5, 30.1)	26.4 (18.4, 35.6)		27.6 (19.7, 36.7)
Rodgers [53]	2005	New Zealand	Text-based	No intervention	6 months	1705 (11.02)	29.0 (26.0, 32.2)	18.8 (16.2, 21.5)	25.4 (22.5, 28.4)		23.7 (20.9, 26.7)
Swartz [54]	2006	United States	Multiplatform	No intervention	3 months	351 (2.27)	12.3 (7.8, 18.2)	5 (2.3, 9.27)	12.3 (7.8, 18.2)		5 (2.3, 9.27)
Liao [55]	2018	China	Text-based	No intervention	6 months	1085 (7.01)	8.3 (6.3, 10.7)	2.2 (1.0, 4.1)	6.8 (5.0, 9.0)		1.9 (0.8, 3.8)
Nguyen [56]	2019	France	Text-based	Self-help guideline	12 months	2478 (16.02)	27.5 (25.1, 30.1)	23.5 (21.1, 25.9)	20.8 (18.6, 23.2)		20.6 (18.3, 22.9)
Bricker [57]	2020	United States	Text-based	Self-help guideline	12 months	2415 (15.61)	14.4 (12.5, 16.5)	7.8 (6.4, 9.5)	9.6 (8.0, 11.3)		5.4 (4.2, 6.8)
Mussenner [58]	2016	Sweden	Text-based	No intervention	3 months	1590 (10.28)	24.5 (21.6, 27.6)	13.8 (11.4, 16.4)	24.5 (21.6, 27.6)		13.8 (11.4, 16.4)
Michele [59]	2012	Turkey	Text-based	Self-help guideline	3 months	151 (0.98)	14.5 (7.5, 24.4)	6.7 (2.2, 14.9)	14.5 (7.5, 24.4)		6.7 (2.2, 14.9)
Mays [60]	2021	United States	Multiplatform	No intervention	6 months	232 (1.49)	38.1 (29.1, 47.7)	11.8 (6.6, 19.0)	51.3 (41.7, 60.8)		27.7 (19.9, 36.7)
Garcia-Pazo [61]	2021	Spain	Multiplatform	Self-help guideline	6 months	110 (0.71)	52.5 (39.3, 65.4)	34.7 (21.7, 49.6)	39.3 (27.1, 52.7)		32.7 (19.9, 47.5)
Chulasai [62]	2022	Thailand	Multiplatform	No intervention	3 months	273 (1.53)	58.4 (49.7, 66.7)	22.1 (15.4, 30.0)	58.4 (49.7, 66.7)		22.1 (15.4, 30.0)
Overall	N/A^d^	N/A	N/A	N/A	N/A	15,472	21.8 (20.9, 22.7)	14.5 (13.7, 15.3)	17.8 (17.0, 18.7)		13.5 (12.7, 14.2)

^a^First author, except for Kraft, who is the second author of the clinical trial. This exception was made because this clinical trial’s first author was also Brendryen, though these 2 trials were completely different samples.

^b^Abstinence is calculated when treatment lost to follow up as relapse.

^c^United States, United Kingdom, Australia, and Singapore.

^d^N/A: not applicable.

### Abstinence

**Figure 2:**
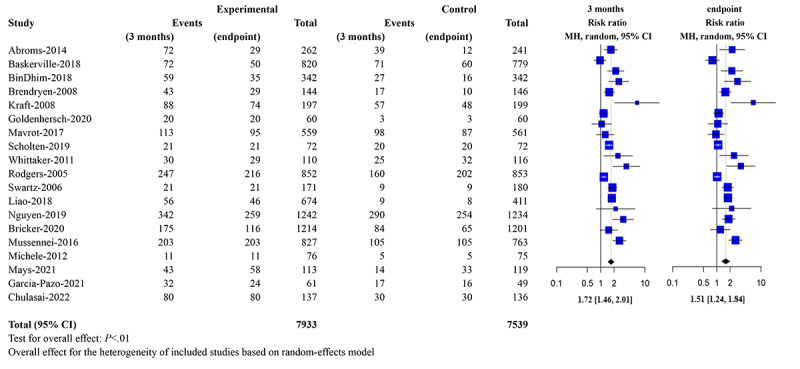
Forest plot of abstinence at 3 months and final follow-up. MH: Mantel-Haenszel method.

Figure 2 [44-62] shows a forest plot of 3-month and final abstinence (for a forest plot of 6-month abstinence, see Supporting Information 4 in Multimedia Appendix 1). For final abstinence, the ADI had a moderate effect compared to controls (RR 1.58, 95% CI 1.31, 1.90). Similarly, for 3-month and 6-month abstinence, ADI also showed a moderate effect relative to controls (RR 1.72, 95% CI 1.46, 2.01) and (RR 1.43, 95% CI 1.17, 1.74), respectively. The sensitivity test demonstrated that the overall effect was strong. Omitting any trial would not change the overall effect significantly in the 3-month results, 6-month results, and endpoint results (for sensitivity test forest plots, see Multimedia Appendix 1: Supporting Information 5.1 for 3-month results; Supporting Information 5.2 for 6-month results; and Supporting Information 5.3 for endpoint results).

### Subgroup Analysis

Subgroup analysis results for 3-month and endpoint abstinence are displayed in Table 2. We divided studies into subgroups by the most commonly used theories (transtheoretical model of behavior change and cognitive behavioral therapy), and there was no evidence that a particular theory or the combination of these theories had a significant effect on intervention efficiency. With respect to sample size, there was no significant difference between small-sample trials (sample size ≤500) and large-sample trials (sample size >500). Similarly, we did not find a significant difference between trials with 2 different controls or 2 different interventions. RR of studies that were compared with no intervention is significantly higher compared with that of self-help guideline. For intervention duration, we did not find any difference between interventions that lasted for more than 1 month and less than 1 month.

**Table 2:** Subgroup analysis of abstinence during the 3-month and endpoint abstinence.

Subgroup		Sample size	Risk ratio (95% CI)	Risk ratio (95% CI)
			3-month	Endpoint
**Strategies used for digital interventions**				
	Both	7187	1.59 (1.32, 1.91)	1.43 (1.12, 1.83)
	Cognitive behavioral therapy	1547	2.99 (1.62, 5.53)	2.30 (1.28, 4.12)
	Transtheoretical model of behavior change	6321	1.48 (1.11, 1.97)	1.43 (1.12, 1.83)
	Others	417	1.70 (0.69, 4.21)	1.65 (1.26, 2.15)
	*P* value for subgroup difference	N/A^a^	.23	.50
**Sample size**				
	>500	13,179	1.54 (1.28, 1.86)	1.41 (1.11, 1.78)
	≤500	2293	1.98 (1.51, 2.60)	1.78 (1.34, 2.37)
	*P* value for subgroup difference	N/A	.13	.21
**Control**				
	Self-help guideline	10,126	1.51 (1.26, 1.80)	1.42 (1.14, 1.76)
	No intervention	5236	2.22 (1.70, 2.91)	1.94 (1.35, 2.78)
	*P* value for subgroup difference	N/A	.02	.14
**Intervention type**				
	Text-based	11,047	1.59 (1.31, 1.92)	1.48 (1.16, 1.89)
	Multiplatform-based	4425	1.85 (1.41, 2.44)	1.61 (1.24, 2.24)
	*P* value for subgroup difference	N/A	.36	.54
**Intervention duration**				
	>1 month	12,308	1.70 (1.41, 2.04)	1.59 (1.28, 1.97)
	≤1 month	3164	1.89 (1.24, 2.90)	1.65 (1.00, 2.71)
	*P* value for subgroup difference	N/A	.64	.89
Overall		15,472	1.72 (1.46, 2.01)	1.58 (1.31, 1.90)

^a^N/A: not applicable.

### Intervention Theories

Tables 3 and 4 report the frequency of trials presenting the TCS items (both items and categories) and the results of univariate regression for abstinence at the endpoint. Most of the items and categories did not show any significant association with intervention efficiency except for I7, I10, and C2. However, only 1 study was coded present in I3 and I10, which was excluded from multivariate metaregression. To avoid collinearity, we did not run multivariate regression with I7 and C2 because I7 is an item inside category 2 (C2) Subsequently, I7 and C2, which pertain to theory-related constructs or predictors, were significantly and independently associated with intervention efficiency. For 3-month and 6-month abstinence, similar results were observed (Supporting Information 6 in Multimedia Appendix 1).

For detailed description of each item and category of the TCS, see Supporting Information 7 in Multimedia Appendix 1.

**Table 3:** Univariate regression of the theory coding scheme (TCS) items, TCS categories, and total score at endpoint (results of metaregression).

Theory coding scheme item (item number)	Studies where item coded as present, n (%)	Univariate		
		Β	95% CI	*P* value
Theory or model of behavior mentioned (I1)	19 (100)	N/A^a^	N/A	N/A
Targeted construct mentioned as predictor of behavior (I2)	15 (89)	0.1505	(–0.5630, 0.8640)	.68
Intervention based on single theory (I3)	2 (11)	0.4335	(–0.2318, 1.0989)	.20
Theory or predictors used to select recipients for the intervention (I4)	1 (5)	0.4489	(–0.6274, 1.5252)	.41
Theory or predictors used to select or develop intervention techniques (I5)	19 (100)	N/A	N/A	N/A
Theory or predictors used to tailor intervention techniques to recipients (I6)	1 (5)	0.4489	(–0.6274, 1.5252)	.41
All intervention techniques are explicitly linked to at least one theory-relevant construct or predictor (I7)	3 (16)	0.6459	(0.0530, 1.2387)	.03^b^
At least one, but not all, of the intervention techniques are explicitly linked to at least one theory-relevant construct or predictor (I8)	18 (95)	0.5413	(–0.3370, 1.4197)	.23
Group of techniques are linked to a group of constructs or predictors (I9)	3 (16)	0.2127	(–0.3329, 0.7583)	.45
All theory-relevant constructs or predictors are explicitly linked to at least one intervention technique (I10)	1 (5)	1.4679	(0.1052, 2.8306)	.03^b^
At least one, but not all, of the theory-relevant constructs or predictors are explicitly linked to at least one intervention technique (I11)	17 (89)	0.5188	(–0.1237, 1.1613)	.11
Theory-relevant constructs are measured: after intervention (I12a)	18 (95)	0.5413	(–0.3370, 1.4197)	.23
Theory-relevant constructs are measured: after and before intervention (I12b)	10 (53)	0.0811	(–0.3403, 0.5025)	.71
Changes in measured theory-relevant constructs or predictors (I13)	9 (47)	0.2884	(–0.1167, 0.6935)	.16
Mediator predicts the dependent variable (I14a)	2 (11)	0.2495	(–0.4229, 0.9218)	.47
Mediator predicts dependent variable, controlling for the independent variable (I14b)	2 (11)	0.2495	(–0.4229, 0.9218)	.47
Intervention does not predict the dependent variable when controlling the independent variable (I14c)	0 (0)	N/A	N/A	N/A
Mediated effect is statistically significant (I14d)	2 (11)	0.2495	(–0.4229, 0.9218)	.47
Results discussed in relation to theory (I15)	8 (42)	0.0104	(–0.4144, 0.4352)	.96
Appropriate support for theory (I16)	2 (11)	0.2495	(–0.4229, 0.9218)	.47
Results used to refine theory: adding or removing constructs to the theory (I17a)	0 (0)	N/A	N/A	N/A
Results used to refine theory: specifying that the interrelationships between the theoretical constructs should be changed (I17b)	0 (0)	N/A	N/A	N/A

^a^N/A: not applicable.

^b^*P*<.05.

**Table 4:** Univariate regression of the theory coding scheme (TCS) items, TCS categories, and included items.

Theory coding scheme categories (category number)	Items included	Univariate		
		Β	95% CI	*P* value
Reference to underpinning theory (C1)	1, 2, 3	0.2683	(–0.1883, 0.7249)	.25
Targeting of relevant theoretical constructs (C2)	2, 5, 6, 7, 8, 9, 10, 11	0.2558	(0.0619, 0.4497)	.01^a^
Using theory to select recipients or tailor interventions (C3)	4, 6	0.2245	(–0.3137, 0.7626)	.41
Measurement of constructs (C4)	12a, 12b	0.1363	(–0.2128, 0.4854)	.44
Testing of theory: mediation effects (C5)	12a, 12b, 13, 14a, 14b, 14c, 14d, 15, 16	0.0524	(–0.0482, 0.1531)	.31
Refining theory (C6)	17a, 17b			
Total use of theory	All items	0.0524	(–0.0068, 0.1116)	.08

^a^*P*<.05.

## Discussion

### Principal Results

To our knowledge, this is the first systematic review and meta-analysis of trials of the effectiveness of ADIs, including both text-based and multiplatform-based trials, on smoking cessation. This meta-analysis provides the latest and strongest evidence on the overall effectiveness of ADIs for smoking cessation by finding that ADIs had a moderate effect (RR 1.58, 95% CI 1.31, 1.90) on smoking cessation, compared to self-help guidelines or to no intervention.

### Comparison With Prior Work

Although plausible evidence from clinical trials that directly compared ADIs and traditional therapies (including pharmacotherapies and psychotherapies) is not available, this moderate effect of ADIs found in the present study is basically comparable with most traditional interventions documented in previous meta-analyses. We extracted the RR with 95% CI from previous meta-analyses, and the RR (95% CI) for endpoint abstinence compared to placebo or no treatment was as follows: RR 2.24, 95% CI (2.06, 2.43) [63]; RR 1.64, 95% CI (1.52, 1.77) for bupropion [64]; and RR 1.60, 95% CI (1.53, 1.68) for NRT [65], among which only varenicline showed a significantly larger RR than ADIs. Regarding the effectiveness of individualized face-to-face psychological interventions on smoking cessation, the most recently updated meta-analysis reported (RR 1.57, 95% CI 1.40, 1.77) as the overall RR (95% CI) of face-to-face psychological interventions compared to minimal interventions (self-help booklet and brief advice) for 6-month abstinence [10].

To compare rates of effectiveness directly, we also calculated the rates (95% CI) of abstinence during the endpoint for the aforementioned meta-analyses. Accordingly, the abstinence rate of the ADIs during the endpoint in this study, which achieved 16.4, 95% CI (15.5, 17.2), was lower than 25.6, 95% CI (24.5, 26.6) of varenicline, comparable to 19.7, 95% CI (8.8, 20.6) and 16.9, 95% CI (16.5, 17.3) for bupropion and NRT, and higher than 10.9, 95% CI (10.1, 11.8) of individualized face-to-face counselling [10,63,64,66]. When compared with pharmacotherapies, the following factors are noteworthy. First, a moderate level of side effects, especially nausea, is reported in all pharmacotherapies, and there is a notable increase in serious adverse effects, including infections and cardiovascular events, with the use of varenicline and NRT [63,65]. Second, an increase in dropouts due to side effects has been reported for bupropion [64]. In addition, participants engaging trials of pharmacotherapies may have more motivation than other interventions because they consented to participate, even though they were made aware in advance of possible side effects. For the comparison with face-to-face psychological interventions, the results of this study are consistent with the results of research on other substance use disorders, as a systematic review demonstrated that there might be no or little difference in effectiveness between digital interventions and face-to-face interventions on lowering alcohol consumption [67].

A systematic review and meta-analysis focused on text-based interventions for smoking cessation was conducted in 2019 [29]. The prior published meta-analysis regarding digital interventions on smoking cessation included both human-delivered interventions and automated-delivered interventions. With the advantages of less professional human resource costs comparing to human-delivered ones [11,12,16,17], the effectiveness of ADIs have not been investigated separately from human-delivered interventions in previous meta-analyses. It documented an RR of 1.54, 95% CI (1.19, 2.00) for final abstinence, which did not significantly differ from that for ADIs found by this study. It is also consistent with the finding of this study that the average RR for final abstinence did not significantly differ from between multiplatform-based (RR 1.48, 95% CI 1.16, 1.89) and text-based (RR 1.61, 95% CI 1.10, 2.35) interventions, but the RR of loss to follow-up of multiplatform-based interventions (RR 0.80, 95% CI 0.59, 1.09) was significantly lower than that of text-based interventions (RR 1.22, 95% CI 1.01, 1.48) at 3 months (*P*=.02), although those during the whole follow-up period (RR 0.87, 95% CI 0.56, 1.36 versus RR 1.18, 95% CI 0.95, 1.47) did not differ significantly (*P*=.06) (Supporting Information 7 in Multimedia Appendix 1). Whether multiplatform-based interventions, such as serious game–based [51] and virtual reality–based [49], may offer advantages in compliance and acceptability associated with the interest of participants needs to be further tested directly, comparing studies with much larger samples. Mixed results were reported when digital interventions were applied as an adjuvant therapy to traditional interventions. An RCT found that abstinence for intervention group (traditional therapies plus digital interventions) was 2.15 times higher than control group (traditional interventions only) in 12-month follow-up (odds ratio=3.13, 95% CI 1.53, 6.71) [68]; however, 2 other trials documented null results [69,70].

Nonetheless, integrating available evidence with the findings of this study, we believe that ADIs could be an effective treatment for smokers. With our estimated effect size, ADIs could increase the abstinence rate by approximately 50% on average. Globally, if available for 20% of the 1.3 billion adult tobacco users who have tried to quit [71] (although most of them failed due to lack of available help), it would help many smokers achieve cessation.

To our knowledge, this study is also the first to assess the empirical evidence of a potential relationship between the effectiveness of digital interventions on smoking cessation and psychological theory. Even though there is a limited number of included studies (ie, 16), which could result in insensitive and underpowered metaregression [72], we found that TCS item I7, “All theory-relevant constructs or predictors are explicitly linked to at least one intervention technique,” was significantly and independently associated with a higher rate of final abstinence. This item pertains to theory-related constructs or predictors, which refer to the theory that digital intervention is not exclusively based on the concept of addictive behaviors but also based on other concepts, such as craving, anxiety, and dependence, which may be more closely linked to the cognitive and affective mechanisms of addiction [3]. Similarly, in a previous meta-analysis on the effectiveness of digital interventions in reducing hazardous alcohol use, the TCS-based metaregression model also found that an item pertaining to theory-related constructs or predictors was significantly associated with better effectiveness [73]. Those findings highlight the importance of theory-related constructs or predictors, especially when developing or optimizing digital interventions for substance use disorders. Based on our findings and previous evidence, studying not only the outcome but also theory-related constructs or predictors offers significant promise for our attempts to unlock what is essentially a black box of mechanisms to understand the theoretical bases and apply that knowledge to improve overall and individualized effectiveness [74-77]. Accordingly, future studies focusing on optimally integrating psychotherapeutic theories and techniques would further increase the efficiency of digital interventions on smoking cessation at both the individualized and overall levels.

### Limitations

We also conducted subgroup analysis to explain the moderate heterogeneity of the studies. However, the results suggested that the analyzed variates were not significantly connected to overall effectiveness. A high rate of loss to follow-up in some studies might contribute to the heterogeneity of this analysis [49]; although we conducted intention-to-treat analysis of the included studies, intention-to-treat analysis could underestimate the results, and complete case analysis could potentially benefit. However, insufficient data limit further analysis. Furthermore, the sensitivity analysis also suggested that any single study could not significantly alter the results. Future studies should enlarge the sample size, which could achieve more satisfactory heterogeneity and provide more accurate results for effect size analysis. Other limitations of this study are also noteworthy. First, the quality of evidence could be a concern because not all the original studies included in this meta-analysis used blind interventions for participants due to feasibility; the risk of bias regarding randomization and intervention delivery might be lowered by the automated procedures of interventions. Second, the self-report abstinence of some studies could bring performance bias to this study, although it would affect both the experimental group and the control group and would unlikely change the effect size measured by RR. Third, most of the included studies were conducted in Western countries, and this could limit the generalization of the results to non-Western countries. Studies conducted in Eastern countries are limited, and we believe that Eastern countries could learn from the experience of previous studies to address the problem of tobacco smoking, as the limited number of studies conducted in Eastern countries are also majorly reported helpful results. In addition, socioeconomic status and age could be potential confounding factors for effectiveness as certain studies were conducted on special populations (such as college students). However, insufficient data made it unable to perform subgroup analysis. For intervention theories, the number of included studies limited the power for detecting some associations, including when assessing differences in how theory is applied.

### Conclusions

Those limitations notwithstanding, this study indicated that ADIs on the transtheoretical model of behavior change and cognitive behavioral therapy had a clear effect compared to self-help guidelines or to no intervention, and effectiveness is associated with some theory-related constructs or predictors. Accordingly, ADIs should be promoted by policy makers and clinical practitioners to fill the huge gap between the need for smoking cessation and treatment resources and should be studied further to increase efficiency by optimally integrating psychotherapeutic theories and techniques.

## Appendix

Multimedia Appendix 1 Supporting information.

## Abbreviations

ADI: automated digital intervention
LMIC: low- and middle-income countries
NRT: nicotine replacement therapy
PRISMA: Preferred Reporting Items for Systematic Reviews and Meta-Analyses
RCT: randomized controlled trial
RR: risk ratio
TCS: theory coding scheme

## References

[1] Peacock A, Leung J, Larney S, Colledge S, Hickman M, Rehm J, Giovino GA, West R, Hall W, Griffiths P, Ali R, Gowing L, Marsden J, Ferrari AJ, Grebely J, Farrell M, Degenhardt L (2018). Global statistics on alcohol, tobacco and illicit drug use: 2017 status report. Addiction.

[2] GBD 2017 Risk Factor Collaborators (2018). Global, regional, and national comparative risk assessment of 84 behavioural, environmental and occupational, and metabolic risks or clusters of risks for 195 countries and territories, 1990-2017: a systematic analysis for the Global Burden of Disease Study 2017. Lancet.

[3] Kalin NH (2020). Substance use disorders and addiction: Mechanisms, trends, and treatment implications. Am J Psychiatry.

[4] West R, Raw M, McNeill A, Stead L, Aveyard P, Bitton J, Stapleton J, McRobbie H, Pokhrel S, Lester-George A, Borland R (2015). Health-care interventions to promote and assist tobacco cessation: a review of efficacy, effectiveness and affordability for use in national guideline development. Addiction.

[5] Prochaska JJ, Benowitz NL (2016). The past, present, and future of nicotine addiction therapy. Annu Rev Med.

[6] Cahill K, Stevens S, Perera R, Lancaster T (2013). Pharmacological interventions for smoking cessation: an overview and network meta-analysis. Cochrane Database Syst Rev.

[7] Fiore MC, Jaén CR (2008). A clinical blueprint to accelerate the elimination of tobacco use. JAMA.

[8] Quik M, Zhang D, Perez XA, Bordia T (2014). Role for the nicotinic cholinergic system in movement disorders; therapeutic implications. Pharmacol Ther.

[9] Stead LF, Buitrago D, Preciado N, Sanchez G, Hartmann-Boyce J, Lancaster T (2013). Physician advice for smoking cessation. Cochrane Database Syst Rev.

[10] Lancaster T, Stead LF (2017). Individual behavioural counselling for smoking cessation. Cochrane Database Syst Rev.

[11] Grant BF, Saha TD, Ruan WJ, Goldstein RB, Chou SP, Jung J, Zhang H, Smith SM, Pickering RP, Huang B, Hasin DS (2016). Epidemiology of DSM-5 drug use disorder: Results from the national epidemiologic survey on alcohol and related conditions-III. JAMA Psychiatry.

[12] Tighe SA, Ball K, Kensing F, Kayser L, Rawstorn JC, Maddison R (2020). Toward a digital platform for the self-management of noncommunicable disease: Systematic review of platform-like interventions. J Med Internet Res.

[13] Choudhry NK, Krumme AA, Ercole PM, Girdish C, Tong AY, Khan NF, Brennan TA, Matlin OS, Shrank WH, Franklin JM (2017). Effect of reminder devices on medication adherence: The REMIND randomized clinical trial. JAMA Intern Med.

[14] Lv Q, Jiang Y, Qi J, Zhang Y, Zhang X, Fang L, Tu L, Yang M, Liao Z, Zhao M, Guo X, Qiu M, Gu J, Lin Z (2019). Using Mobile Apps for Health Management: A New Health Care Mode in China. JMIR Mhealth Uhealth.

[15] Fu Z, Burger H, Arjadi R, Bockting CLH (2020). Effectiveness of digital psychological interventions for mental health problems in low-income and middle-income countries: a systematic review and meta-analysis. Lancet Psychiatry.

[16] Carson KV, Verbiest MEA, Crone MR, Brinn MP, Esterman AJ, Assendelft WJJ, Smith BJ (2012). Training health professionals in smoking cessation. Cochrane Database Syst Rev.

[17] Hasin DS, O'Brien CP, Auriacombe M, Borges G, Bucholz K, Budney A, Compton WM, Crowley T, Ling W, Petry NM, Schuckit M, Grant BF (2013). DSM-5 criteria for substance use disorders: recommendations and rationale. Am J Psychiatry.

[18] Byrne S, Kotze B, Ramos F, Casties A, Harris A (2020). Using a mobile health device to manage severe mental illness in the community: What is the potential and what are the challenges?. Aust N Z J Psychiatry.

[19] Källander K, Tibenderana JK, Akpogheneta OJ, Strachan DL, Hill Z, ten Asbroek AHA, Conteh L, Kirkwood BR, Meek SR (2013). Mobile health (mHealth) approaches and lessons for increased performance and retention of community health workers in low- and middle-income countries: a review. J Med Internet Res.

[20] Mishra SR, Lygidakis C, Neupane D, Gyawali B, Uwizihiwe JP, Virani SS, Kallestrup P, Miranda JJ (2019). Combating non-communicable diseases: potentials and challenges for community health workers in a digital age, a narrative review of the literature. Health Policy Plan.

[21] Schwalm J, McCready T, Lopez-Jaramillo P, Yusoff K, Attaran A, Lamelas P, Camacho PA, Majid F, Bangdiwala SI, Thabane L, Islam S, McKee M, Yusuf S (2019). A community-based comprehensive intervention to reduce cardiovascular risk in hypertension (HOPE 4): a cluster-randomised controlled trial. Lancet.

[22] Quinn CC, Shardell MD, Terrin ML, Barr EA, Ballew SH, Gruber-Baldini AL (2011). Cluster-randomized trial of a mobile phone personalized behavioral intervention for blood glucose control. Diabetes Care.

[23] Young HM, Miyamoto S, Dharmar M, Tang-Feldman Y (2020). Nurse Coaching and Mobile Health Compared With Usual Care to Improve Diabetes Self-Efficacy for Persons With Type 2 Diabetes: Randomized Controlled Trial. JMIR Mhealth Uhealth.

[24] Faurholt-Jepsen M, Bauer M, Kessing LV (2018). Smartphone-based objective monitoring in bipolar disorder: status and considerations. Int J Bipolar Disord.

[25] Xu DR, Xiao S, He H, Caine ED, Gloyd S, Simoni J, Hughes JP, Nie J, Lin M, He W, Yuan Y, Gong W (2019). Lay health supporters aided by mobile text messaging to improve adherence, symptoms, and functioning among people with schizophrenia in a resource-poor community in rural China (LEAN): A randomized controlled trial. PLoS Med.

[26] Litvin EB, Abrantes AM, Brown RA (2013). Computer and mobile technology-based interventions for substance use disorders: an organizing framework. Addict Behav.

[27] Naslund JA, Aschbrenner KA, Araya R, Marsch LA, Unützer J, Patel V, Bartels SJ (2017). Digital technology for treating and preventing mental disorders in low-income and middle-income countries: a narrative review of the literature. Lancet Psychiatry.

[28] Bennett KM, Clary KL, Smith DC, Lee CA (2020). Usability and Acceptability of a Mobile App to Help Emerging Adults Address their Friends' Substance Use (Harbor): Quantitative Study. J Med Internet Res.

[29] Whittaker R, McRobbie H, Bullen C, Rodgers A, Gu Y, Dobson R (2019). Mobile phone text messaging and app-based interventions for smoking cessation. Cochrane Database Syst Rev.

[30] Prestwich A, Sniehotta FF, Whittington C, Dombrowski SU, Rogers L, Michie S (2014). Does theory influence the effectiveness of health behavior interventions? Meta-analysis. Health Psychol.

[31] Jia Y, Fu H, Gao J, Dai J, Zheng P (2018). The roles of health culture and physical environment in workplace health promotion: a two-year prospective intervention study in China. BMC Public Health.

[32] Chu AHY, Ng SHX, Tan CS, Win AM, Koh D, Müller-Riemenschneider F (2016). A systematic review and meta-analysis of workplace intervention strategies to reduce sedentary time in white-collar workers. Obes Rev.

[33] Gardner B, Whittington C, McAteer J, Eccles MP, Michie S (2010). Using theory to synthesise evidence from behaviour change interventions: the example of audit and feedback. Soc Sci Med.

[34] Painter JE, Borba CPC, Hynes M, Mays D, Glanz K (2008). The use of theory in health behavior research from 2000 to 2005: a systematic review. Ann Behav Med.

[35] Webb TL, Joseph J, Yardley L, Michie S (2010). Using the internet to promote health behavior change: a systematic review and meta-analysis of the impact of theoretical basis, use of behavior change techniques, and mode of delivery on efficacy. J Med Internet Res.

[36] Noar SM, Benac CN, Harris MS (2007). Does tailoring matter? Meta-analytic review of tailored print health behavior change interventions. Psychol Bull.

[37] Higgins JPT, Thompson SG, Deeks JJ, Altman DG (2003). Measuring inconsistency in meta-analyses. BMJ.

[38] Glanz K, Bishop DB (2010). The role of behavioral science theory in development and implementation of public health interventions. Annu Rev Public Health.

[39] Gardner B, Wardle J, Poston L, Croker H (2011). Changing diet and physical activity to reduce gestational weight gain: a meta-analysis. Obes Rev.

[40] Moher D, Liberati A, Tetzlaff J, Altman DG, PRISMA Group (2009). Preferred reporting items for systematic reviews and meta-analyses: the PRISMA statement. PLoS Med.

[41] Higgins JPT, Altman DG, Gøtzsche PC, Jüni P, Moher D, Oxman AD, Savovic J, Schulz KF, Weeks L, Sterne JAC, Cochrane Bias Methods Group, Cochrane Statistical Methods Group (2011). The Cochrane Collaboration's tool for assessing risk of bias in randomised trials. BMJ.

[42] Michie S, Prestwich A (2010). Are interventions theory-based? Development of a theory coding scheme. Health Psychol.

[43] Harbord RM, Egger M, Sterne JAC (2006). A modified test for small-study effects in meta-analyses of controlled trials with binary endpoints. Stat Med.

[44] Abroms LC, Boal AL, Simmens SJ, Mendel JA, Windsor RA (2014). A randomized trial of Text2Quit: a text messaging program for smoking cessation. Am J Prev Med.

[45] Baskerville NB, Struik LL, Guindon GE, Norman CD, Whittaker R, Burns C, Hammond D, Dash D, Brown KS (2018). Effect of a Mobile Phone Intervention on Quitting Smoking in a Young Adult Population of Smokers: Randomized Controlled Trial. JMIR Mhealth Uhealth.

[46] BinDhim NF, McGeechan K, Trevena L (2018). Smartphone Smoking Cessation Application (SSC App) trial: a multicountry double-blind automated randomised controlled trial of a smoking cessation decision-aid 'app'. BMJ Open.

[47] Brendryen H, Drozd F, Kraft P (2008). A digital smoking cessation program delivered through internet and cell phone without nicotine replacement (happy ending): randomized controlled trial. J Med Internet Res.

[48] Brendryen H, Kraft P (2008). Happy ending: a randomized controlled trial of a digital multi-media smoking cessation intervention. Addiction.

[49] Goldenhersch E, Thrul J, Ungaretti J, Rosencovich N, Waitman C, Ceberio MR (2020). Virtual Reality Smartphone-Based Intervention for Smoking Cessation: Pilot Randomized Controlled Trial on Initial Clinical Efficacy and Adherence. J Med Internet Res.

[50] Mavrot C, Stucki I, Sager F, Etter J (2017). Efficacy of an Internet-based, individually tailored smoking cessation program: A randomized-controlled trial. J Telemed Telecare.

[51] Scholten H, Luijten M, Granic I (2019). A randomized controlled trial to test the effectiveness of a peer-based social mobile game intervention to reduce smoking in youth. Dev Psychopathol.

[52] Whittaker R, Dorey E, Bramley D, Bullen C, Denny S, Elley CR, Maddison R, McRobbie H, Parag V, Rodgers A, Salmon P (2011). A theory-based video messaging mobile phone intervention for smoking cessation: randomized controlled trial. J Med Internet Res.

[53] Rodgers A, Corbett T, Bramley D, Riddell T, Wills M, Lin R, Jones M (2005). Do u smoke after txt? Results of a randomised trial of smoking cessation using mobile phone text messaging. Tob Control.

[54] Swartz LHG, Noell JW, Schroeder SW, Ary DV (2006). A randomised control study of a fully automated internet based smoking cessation programme. Tob Control.

[55] Liao Y, Wu Q, Kelly BC, Zhang F, Tang Y, Wang Q, Ren H, Hao Y, Yang M, Cohen J, Tang J (2018). Effectiveness of a text-messaging-based smoking cessation intervention ("Happy Quit") for smoking cessation in China: A randomized controlled trial. PLoS Med.

[56] Nguyen Thanh V, Guignard R, Lancrenon S, Bertrand C, Delva C, Berlin I, Pasquereau A, Arwidson P (2019). Effectiveness of a Fully Automated Internet-Based Smoking Cessation Program: A Randomized Controlled Trial (STAMP). Nicotine Tob Res.

[57] Bricker JB, Watson NL, Mull KE, Sullivan BM, Heffner JL (2020). Efficacy of Smartphone Applications for Smoking Cessation: A Randomized Clinical Trial. JAMA Intern Med.

[58] Müssener U, Bendtsen M, Karlsson N, White IR, McCambridge J, Bendtsen P (2016). Effectiveness of Short Message Service Text-Based Smoking Cessation Intervention Among University Students: A Randomized Clinical Trial. JAMA Intern Med.

[59] Ybarra M, Bağci Bosi AT, Korchmaros J, Emri S (2012). A text messaging-based smoking cessation program for adult smokers: randomized controlled trial. J Med Internet Res.

[60] Mays D, Johnson AC, Phan L, Sanders C, Shoben A, Tercyak KP, Wagener TL, Brinkman MC, Lipkus IM (2021). Tailored mobile messaging intervention for waterpipe tobacco cessation in young adults: A randomized trial. Am J Public Health.

[61] García-Pazo P, Sesé A, Llabrés J, Fornés-Vives J (2021). NoFumo+: A clinical trial of an mHealth for smoking cessation with hospitalized patients. Int J Environ Res Public Health.

[62] Chulasai P, Chinwong D, Vientong P, Lertsinudom S, Kanjanarat P, Hall JJ, Chinwong S (2022). Smartphone application for smoking cessation (Quit with US): A randomized controlled trial among young adult light smokers in Thailand. Int J Environ Res Public Health.

[63] Cahill K, Lindson-Hawley N, Thomas KH, Fanshawe TR, Lancaster T (2016). Nicotine receptor partial agonists for smoking cessation. Cochrane Database Syst Rev.

[64] Howes S, Hartmann-Boyce J, Livingstone-Banks J, Hong B, Lindson N (2020). Antidepressants for smoking cessation. Cochrane Database Syst Rev.

[65] Stead LF, Perera R, Bullen C, Mant D, Hartmann-Boyce J, Cahill K, Lancaster T (2012). Nicotine replacement therapy for smoking cessation. Cochrane Database Syst Rev.

[66] Hartmann-Boyce J, Chepkin SC, Ye W, Bullen C, Lancaster T (2018). Nicotine replacement therapy versus control for smoking cessation. Cochrane Database Syst Rev.

[67] Kaner EF, Beyer FR, Garnett C, Crane D, Brown J, Muirhead C, Redmore J, O'Donnell A, Newham JJ, de Vocht F, Hickman M, Brown H, Maniatopoulos G, Michie S (2017). Personalised digital interventions for reducing hazardous and harmful alcohol consumption in community-dwelling populations. Cochrane Database Syst Rev.

[68] Carrasco-Hernandez L, Jódar-Sánchez F, Núñez-Benjumea F, Moreno Conde J, Mesa González M, Civit-Balcells A, Hors-Fraile S, Parra-Calderón CL, Bamidis PD, Ortega-Ruiz F (2020). A Mobile Health Solution Complementing Psychopharmacology-Supported Smoking Cessation: Randomized Controlled Trial. JMIR Mhealth Uhealth.

[69] Vidrine DJ, Frank-Pearce SG, Vidrine JI, Tahay PD, Marani SK, Chen S, Yuan Y, Cantor SB, Prokhorov AV (2019). Efficacy of Mobile Phone-Delivered Smoking Cessation Interventions for Socioeconomically Disadvantaged Individuals: A Randomized Clinical Trial. JAMA Intern Med.

[70] Wetter DW, McClure JB, Cofta-Woerpel L, Costello TJ, Reitzel LR, Businelle MS, Cinciripini PM (2011). A randomized clinical trial of a palmtop computer-delivered treatment for smoking relapse prevention among women. Psychol Addict Behav.

[71] Asma S, Mackay J, Song SY, Zhao L, Morton J, Palipudi KM The GATS Atlas.

[72] López-López JA, Marín-Martínez F, Sánchez-Meca J, Van den Noortgate W, Viechtbauer W (2014). Estimation of the predictive power of the model in mixed-effects meta-regression: A simulation study. Br J Math Stat Psychol.

[73] Garnett C, Crane D, Brown J, Kaner E, Beyer F, Muirhead C, Hickman M, Redmore J, de Vocht F, Beard E, Michie S (2018). Reported Theory Use by Digital Interventions for Hazardous and Harmful Alcohol Consumption, and Association With Effectiveness: Meta-Regression. J Med Internet Res.

[74] Blalock DV, Calhoun PS, Crowley MJ, Dedert EA (2019). Telehealth Treatment for Alcohol Misuse: Reviewing Telehealth Approaches to Increase Engagement and Reduce Risk of Alcohol-Related Hypertension. Curr Hypertens Rep.

[75] Heinz A, Deserno L, Zimmermann US, Smolka MN, Beck A, Schlagenhauf F (2017). Targeted intervention: Computational approaches to elucidate and predict relapse in alcoholism. Neuroimage.

[76] Brown B, Gude WT, Blakeman T, van der Veer SN, Ivers N, Francis JJ, Lorencatto F, Presseau J, Peek N, Daker-White G (2019). Clinical Performance Feedback Intervention Theory (CP-FIT): a new theory for designing, implementing, and evaluating feedback in health care based on a systematic review and meta-synthesis of qualitative research. Implement Sci.

[77] Hysong SJ, Kell HJ, Petersen LA, Campbell BA, Trautner BW (2017). Theory-based and evidence-based design of audit and feedback programmes: examples from two clinical intervention studies. BMJ Qual Saf.

