# Supplementary material for: Automated Digital Interventions and Smoking Cessation: Systematic Review and Meta-analysis Relating Efficiency to a Psychological Theory of Intervention Perspective
Source: J Med Internet Res. 2022 Nov 16;24(11):e38206. doi: 10.2196/38206 (PMC9713619; doi:10.2196/38206)

Supporting Information 1: full search strategy 2
Supporting Information 2: funnel plot 2
Supporting Information 3: Cochrane ROB2 summary table 3
Supporting Information 4: forest plot of abstinence at 6-month 4
Supporting Information 5: sensitivity forest plot 5
Supporting Information 5.1: sensitivity forest plot of 3-month abstinence 5
Supporting Information 5.2: sensitivity forest plot of 6-month abstinence 6
Supporting Information 5.3: ensitivity forest plot of final follow-up abstinence 7
Supporting Information 6 univariate regression results 8
Supporting Information 6.1 univariate regression of the TCS items, TCS categories and total score at 3-month 8
Supporting Information 6.2 univariate regression of the TCS items, TCS categories and total score at 6-month 9
Supporting Information 7 description of each item and category of the TCS 11
Supporting Information 8 forest plot of lost to follow-up (subgroups by intervention type) 13
Supporting Information 8.1 forest plot of lost to follow-up at 3-month (subgroups by intervention type) 13
Supporting Information 8.2 forest plot of lost to follow-up at final follow-up (subgroups by intervention type) 14

Supporting Information 1: full search strategy

1.digital therapy
#1 digital*[Title/Abstract]
#2 smartphone[Title/Abstract]
#3 smart-phone[Title/Abstract]
#4 computer*[Title/Abstract]
#5 mobile*[Title/Abstract]
#6 cellphone[Title/Abstract]
#7 app*[Title/Abstract]
#8 “mobile health” [Title/Abstract]
#9 telemedicine[Mesh Terms]
#10=#1 OR #2 OR #3 OR #4 OR #5 OR #6 OR #7 OR #8 OR #9

2.tobacco use disorder
#1 tobacco use disorder[Mesh Terms]
#2 nicotine addiction[Title/Abstract]
#3 tobacco dependence[Title/Abstract]
#4 smoking cessation[Mesh Terms]
#5=#1 OR #2 OR #3 OR #4

Results:
Pubmed:1147
Cochrane:2849
Embase:2601
CNKI:17

Supporting Information 2: funnel plot

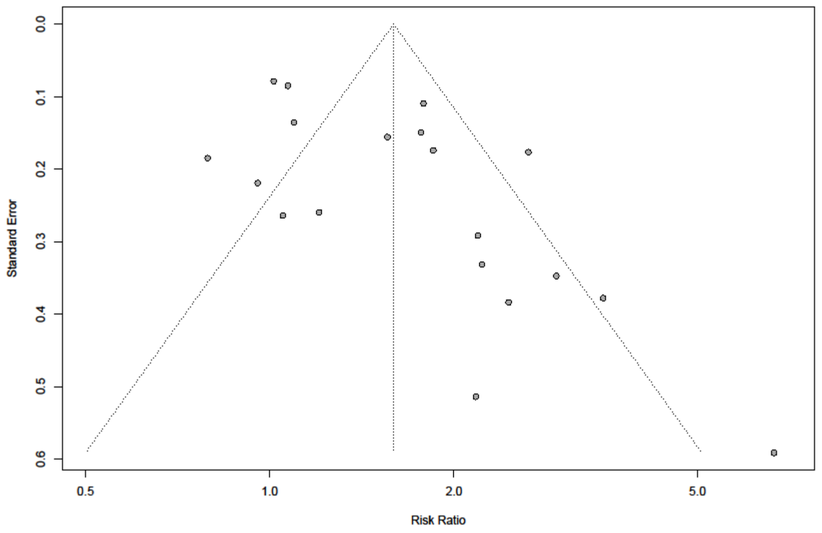

p-value = 0.1318

Supporting Information 3: Cochrane ROB2 summary table

	Randomization process	Deviations from intended interventions	Missing outcome data	Measurement of the outcome	Selection of the reported result	Overall Bias
**Abroms 2014**	Low	Low	Low	Low	Low	Low
**Baskerville 2018**	Low	Low	High	Low	Low	High
**BinDhim 2018**	Low	Low	Low	Low	Low	Low
**Brendryen 2008**	Low	Low	Low	Low	Low	Low
**Kraft 2008**	Low	Low	Low	Low	Low	Low
**Goldenhersc 2020**	Low	Low	High	Low	Low	High
**Mavrot 2017**	Low	Low	High	Low	Low	Some concerns
**Scholten 2019**	Low	Low	Low	Low	Low	Low
**Whittaker 2011**	Low	Low	Some concerns	Low	Low	Some concerns
**Rodgers2005**	Low	Low	Low	Low	Low	Low
**Swartz 2006**	Low	Low	High	Low	Low	High
**Liao 2018**	Low	Low	Low	Low	Low	Low
**Nguyen 2019**	Low	Low	High	Low	Low	High
**Bricker 2020**	Low	Low	Low	Low	Low	Low
**Mussennei 2016**	Low	Low	Low	Low	Low	Low
**Michele 2012**	Low	Low	High	Low	Low	High
**Mays 2021**	Low	Low	Low	Low	Low	Low
**Garcia-Pazo 2021**	Low	Low	Some concerns	Low	Low	Some concerns
**Chulasai 2022**	Low	Low	Low	Low	Low	Low

Supporting Information 4: forest plot of abstinence at 6-month

Study	Experimental Events	Experimental Total	Control Events	Control Total	Weight	Risk Ratio MH, Random, 95% CI
Abroms−2014	29	262	12	241	5.1%	2.22 [1.16, 4.26]
Baskerville−2018	50	820	60	779	8.2%	0.79 [0.55, 1.14]
BinDhim−2018	35	342	16	342	5.8%	2.19 [1.23, 3.88]
Brendryen−2008	34	144	10	146	4.9%	3.45 [1.77, 6.71]
Kraft−2008	73	197	43	199	8.7%	1.71 [1.24, 2.36]
Whittaker−2011	29	110	32	116	7.4%	0.96 [0.62, 1.47]
Bricker−2020	265	1214	158	1201	10.3%	1.66 [1.39, 1.99]
Rodgers−2005	216	852	202	853	10.4%	1.07 [0.91, 1.26]
Nguyen−2019	307	1242	305	1236	10.7%	1.00 [0.87, 1.15]
Liao−2018	46	674	8	411	4.4%	3.51 [1.67, 7.35]
Marvot−2017	95	559	87	561	9.3%	1.10 [0.84, 1.43]
Mays−2021	58	113	33	119	8.4%	1.85 [1.32, 2.60]
Garcia−Pazo−2021	24	61	16	49	6.5%	1.20 [0.72, 2.00]
**Total (95% CI)**		**6590**		**6253**	**100.0%**	**1.43 [1.17, 1.74]**

Heterogeneity: $Tau^2 = 0.091$; $Chi^2 = 63.40$, df = 12 ($P < 0.01$); $I^2 = 81\%$
Test for overall effect: $Z = 3.55$ ($P < 0.01$)

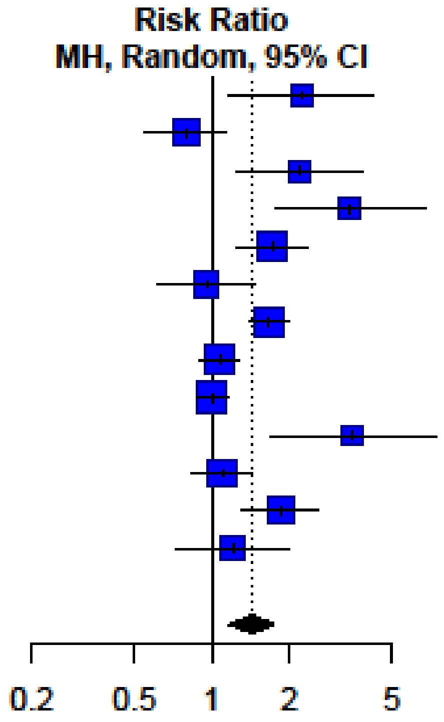

Supporting Information 5: sensitivity forest plot

Supporting Information 5.1: sensitivity forest plot of 3-month abstinence

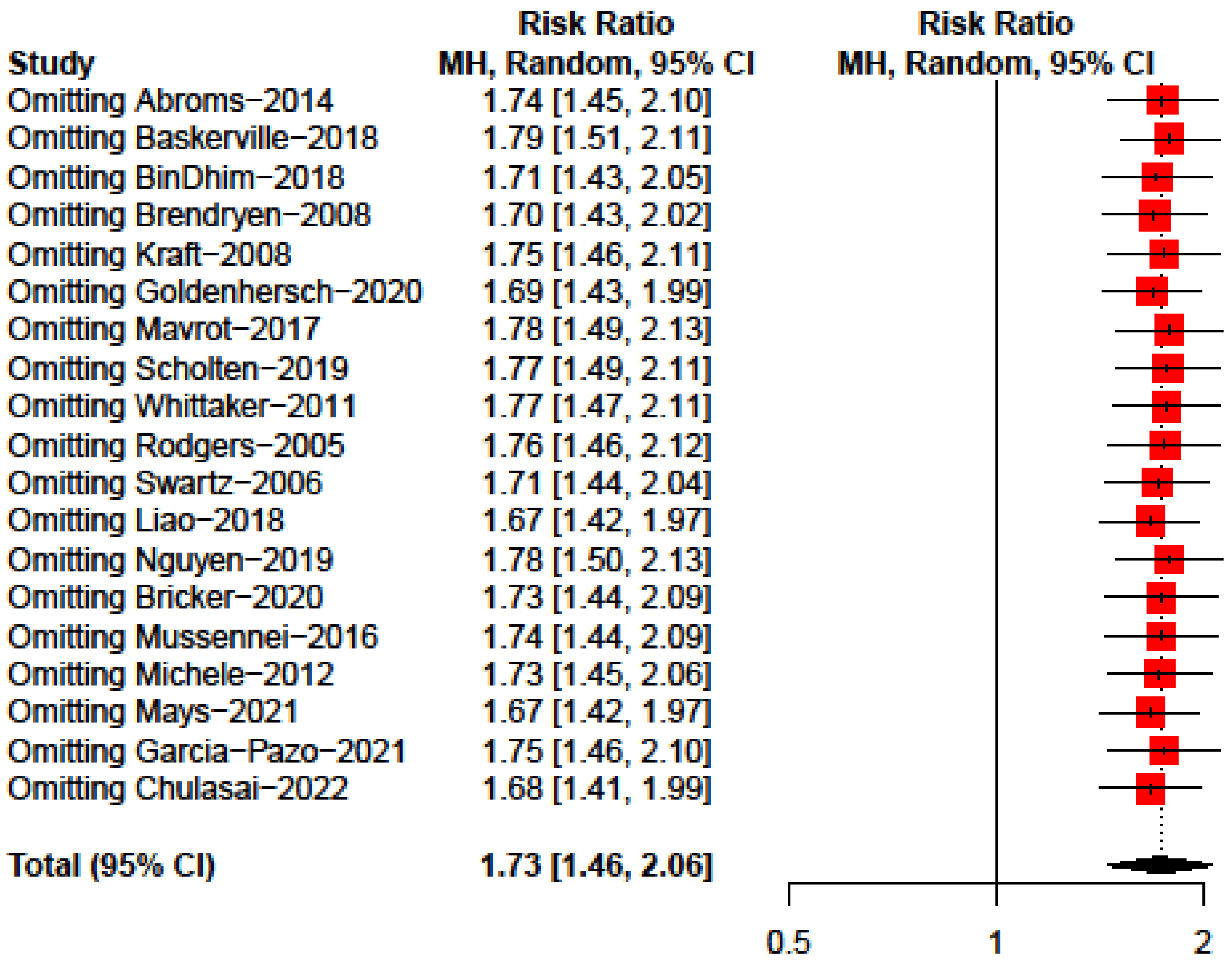

Supporting Information 5.2: sensitivity forest plot of 6-month abstinence

Study	Risk Ratio MH, Random, 95% CI
Omitting Abroms−2014	1.40 [1.14, 1.71]
Omitting Baskerville−2018	1.51 [1.23, 1.84]
Omitting BinDhim−2018	1.39 [1.14, 1.70]
Omitting Brendryen−2008	1.36 [1.12, 1.65]
Omitting Kraft−2008	1.41 [1.14, 1.73]
Omitting Whittaker−2011	1.48 [1.20, 1.82]
Omitting Bricker−2020	1.41 [1.14, 1.73]
Omitting Rodgers−2005	1.49 [1.19, 1.87]
Omitting Nguyen−2019	1.50 [1.21, 1.86]
Omitting Liao−2018	1.37 [1.13, 1.66]
Omitting Marvot−2017	1.48 [1.19, 1.84]
Omitting Mays−2021	1.40 [1.14, 1.71]
Omitting Garcia−Pazo−2021	1.45 [1.18, 1.79]
**Total (95% CI)**	**1.43 [1.17, 1.74]**

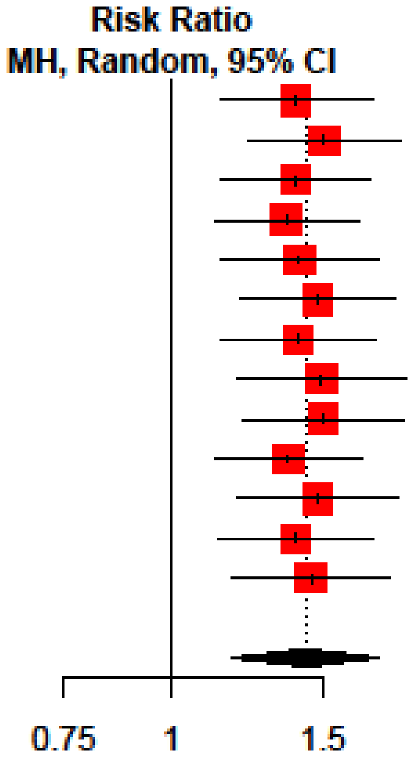

Supporting Information 5.3: Sensitivity forest plot of endpoint abstinence

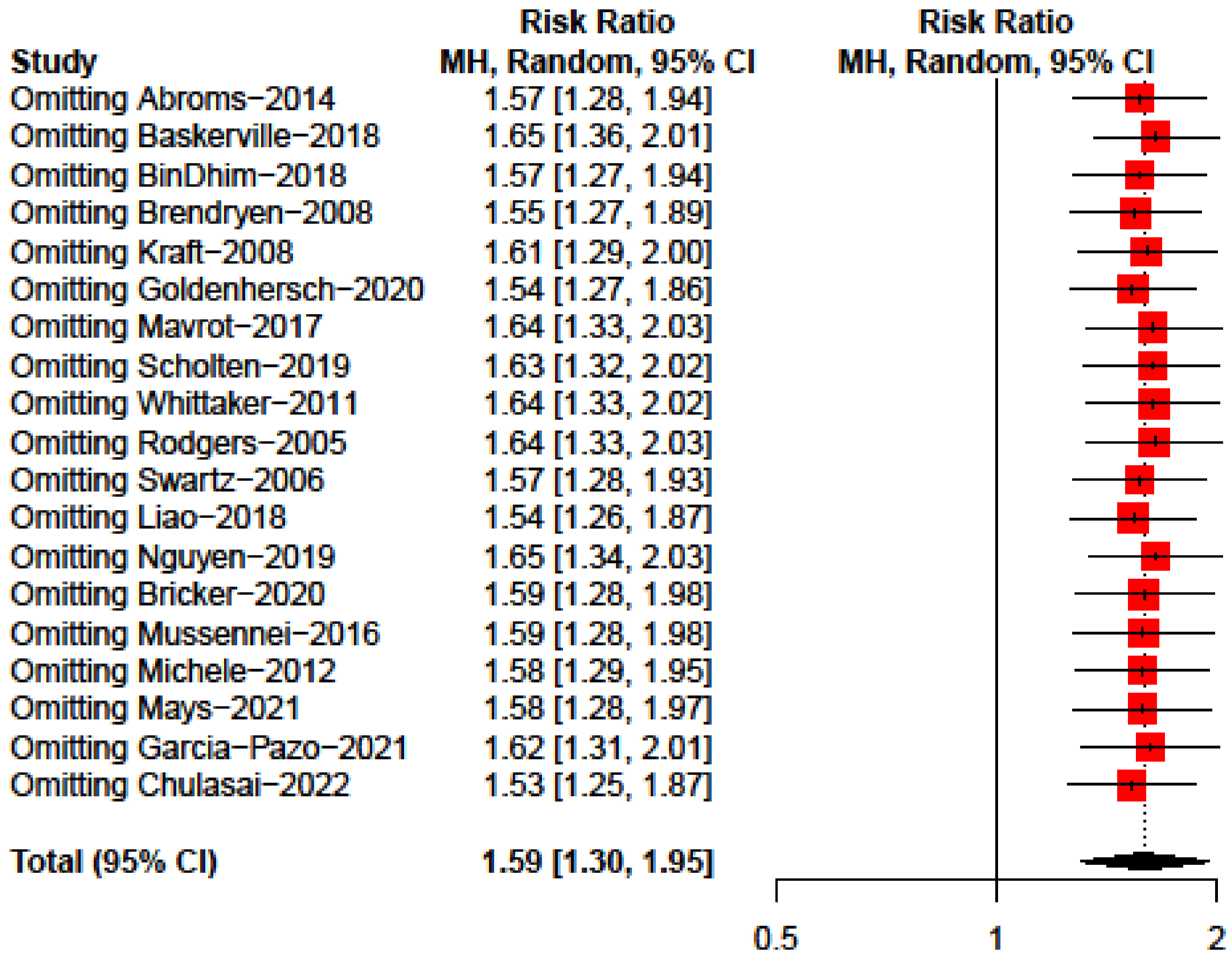

Supporting Information 6 univariate regression results

Supporting Information 6.1 univariate regression of the TCS items, TCS categories and total score at 3-month

Theory coding scheme item (item number)	Item description	Studies where item coded as present, n (%)	Univariate		
			B	95%CI	p
Theory or model of behavior mentioned(I1)	Models or theories that specify relations among variables to explain or predict behavior are mentioned even if the intervention is not based on this theory	19(100)	/	/	/
Targeted construct mentioned as predictor of behavior (I2)	"Targeted" construct refers to a psychological construct that the study intervention is hypothesized to change	15(89)	0.0628	(-0.5317, 0.6573)	0.8359
Intervention based on single theory (I3)	The intervention is based on a single theory (rather than a combination of theories or theory and predictors)	2(11)	0.7665	(0.1981, 1.3349)	0.0082*
Theory or predictors used to select recipients for the intervention (I4)	Participants were screened or selected based on achieving a particular score or level on a theory-relevant construct or predictor	1(5)	0.3701	(-0.5705, 1.3107)	0.4406
Theory or predictors used to select or develop intervention techniques (I5)	The intervention is explicitly based on a theory or predictor or combination of theories and predictors	19(100)	/	/	/
Theory or predictors used to tailor intervention techniques to recipients (I6)	The intervention differs for different subgroups that vary on a psychological construct or predictor at baseline	1(5)	0.3701	(-0.5705, 1.3107)	0.4406
All intervention techniques are explicitly linked to at least one theory-relevant construct or predictor (I7)	Each intervention technique is explicitly linked to at least one theory-relevant construct or predictor	3(16)	0.8914	(0.3799, 1.4030)	0.0006*
At least one, but not all, of the intervention techniques are explicitly linked to at least one theory-relevant construct or predictor (I8)	At least one, but not all, of the intervention techniques are explicitly linked to at least one theory-relevant construct or predictor	18(95)	0.3210	(-0.4168, 1.0589)	0.3938
Group of techniques are linked to a group of constructs or predictors (I9)	A cluster of techniques is linked to a cluster of constructs or predictors	3(16)	0.1103	(-0.3006, 0.5212)	0.5988
All theory-relevant constructs or predictors are explicitly linked to at least one intervention technique (I10)	Every theoretical construct within a state theory, or every stated predictor, is linked to at least one intervention technique	1(5)	1.3819	(0.1034, 2.6604)	0.0341*
At least one, but not all, of the theory-relevant constructs or predictors are explicitly linked to at least one intervention technique (I11)	At least one, but not all, of the theoretical constructs within a stated theory or at least one, but not all, of the stated predictors (see I5) are linked to at least one intervention technique	17(89)	0.4330	(-0.1100, 0.9759)	0.1181
Theory-relevant constructs are measured: post intervention (I12a)	At least one construct of theory (or predictor) mentioned in relation to the intervention is measured post intervention	18(95)	0.3210	(-0.4168, 1.0589)	0.3938
Theory-relevant constructs are measured: post and pre intervention (I12b)	At least one construct of theory (or predictor) mentioned in relation to the intervention is measured pre and post intervention	10(53)	0.0913	(-0.2369, 0.4195)	0.5856
Changes in measured theory-relevant constructs or predictors (I13)	The intervention leads to significant change in at least one theory relevant construct or predictor (vs control group) in favor of the intervention	9(47)	0.2451	(-0.0564, 0.5466)	0.1111
Mediator predicts the dependent variable (I14a)	Mediator predicts dependent variable, or change in mediator leads to change in dependent variable	2(11)	0.1150	(-0.3947, 0.6246)	0.6584
Mediator predicts dependent variable, controlling for the independent variable (I14b)	Mediator predicts dependent variable when controlling for independent variable	2(11)	0.1150	(-0.3947, 0.6246)	0.6584
Intervention does not predict the dependent variable when controlling the independent variable (I14c)	Intervention does not predict dependent variable when controlling for mediator	0(0)	/	/	/
Mediated effect is statistically significant (I14d)	Mediated effect is statistically significant	2(11)	0.1150	(-0.3947, 0.6246)	0.6584

Results discussed in relation to theory (I15)	Results are discussed in terms of the theoretical basis of the intervention	8(42)	0.0636	(-0.2676, 0.3948)	0.7065
Appropriate support for theory (I16)	Support for the theory is based on appropriate mediation, or refutation of the theory is based on obtaining appropriate null effects (i.e., changing behavior without changing the theory-relevant constructs)	2(11)	0.1150	(-0.3947, 0.6246)	0.6584
Results used to refine theory: adding or removing constructs to the theory (I17a)	Authors attempt to refine the theory upon which the intervention was based by adding or removing constructs to the theory	0(0)	/	/	/
Results used to refine theory: specifying that the interrelationships between the theoretical constructs should be changed(I17b)	Authors attempt to refine the theory upon which the intervention was based by specifying that the interrelationships between the theoretical constructs should be changed and spelling out which relationships should be changed	0(0)	/	/	/

**Theory coding scheme categories (category number)**	**Category description**	**Items included**	**univariate**		
			**B**	**95%CI**	**p**
Reference to underpinning theory (C1)	Stated or suggested, rather than demonstrated theoretical base	1, 2, 3	0.3735	(-0.0231, 0.7700)	0.0649
Targeting of relevant theoretical constructs (C2)	Whether evidence was provided that a targeted theoretical construct predicted behavior, whether theory or predictors were explicitly used for designing the intervention, and the extent to which the intervention targets particular theory-relevant constructs	2, 5, 6, 7, 8, 9, 10, 11	0.2176	(0.0492, 0.3859)	0.0113*
Using theory to select recipients or tailor interventions (C3)	Whether theory was used to select participants likely to benefit from the intervention, or to tailor the intervention to the needs of a particular individual	4, 6	0.1851	(-0.2852, 0.6554)	0.4406
Measurement of constructs (C4)	Whether the relevant theory-based constructs or predictors have been measured	12a, 12b	0.1106	(-0.1689, 0.3900)	0.4380
Testing of theory: mediation effects (C5)	Whether theoretical constructs are measured, whether the intervention changes the theoretical constructs, and whether these changes explain the effect	12a, 12b, 13, 14a, 14b, 14c, 14d, 15, 16	0.0366	(-0.0398, 0.1129)	0.3477
Refining theory (C6)	Whether the results of evaluating theory-based interventions are used to refine theory	17a, 17b	/	/	/
Total use of theory	/	All items	0.0413	(-0.0066, 0.0892)	0.0908

* p-value < 0.05

Supporting Information 6.2 univariate regression of the TCS items, TCS categories and total score at 6-month

**Theory coding scheme item (item number)**	**Item description**	**Studies where item coded as present, n (%)**	**Univariate**		
			**B**	**95%CI**	**p**
Theory or model of behavior mentioned(I1)	Models or theories that specify relations among variables to explain or predict behavior are mentioned even if the intervention is not based on this theory	19(100)	/	/	/
Targeted construct mentioned as predictor of behavior (I2)	“Targeted” construct refers to a psychological construct that the study intervention is hypothesized to change	15(89)	0.3447	(-0.2362, 0.9255)	0.2449
Intervention based on single theory (I3)	The intervention is based on a single theory (rather than a combination of theories or theory and predictors)	2(11)	0.9416	(-0.0026, 1.8859)	0.0506
Theory or predictors used to select recipients for the intervention (I4)	Participants were screened or selected based on achieving a particular score or level on a theory-relevant construct or predictor	1(5)	/	/	/
Theory or predictors used to select or develop intervention techniques (I5)	The intervention is explicitly based on a theory or predictor or combination of theories and predictors	19(100)	/	/	/
Theory or predictors used to tailor intervention techniques to recipients (I6)	The intervention differs for different subgroups that vary on a psychological construct or predictor at baseline	1(5)	/	/	/
All intervention techniques are explicitly linked to at least	Each intervention technique is explicitly linked to at least one theory-relevant construct or	3(16)	0.3215	(-0.1659, 0.8089)	0.1961

one theory-relevant construct or predictor (I7)	predictor				
At least one, but not all, of the intervention techniques are explicitly linked to at least one theory-relevant construct or predictor (I8)	At least one, but not all, of the intervention techniques are explicitly linked to at least one theory-relevant construct or predictor	18(95)	0.1152	(-0.3711, 0.6015)	0.6425
Group of techniques are linked to a group of constructs or predictors (I9)	A cluster of techniques is linked to a cluster of constructs or predictors	3(16)	0.4094	(0.0134, 0.8054)	0.0427*
All theory-relevant constructs or predictors are explicitly linked to at least one intervention technique (I10)	Every theoretical construct within a state theory, or every stated predictor, is linked to at least one intervention technique	1(5)	/	/	/
At least one, but not all, of the theory-relevant constructs or predictors are explicitly linked to at least one intervention technique (I11)	At least one, but not all, of the theoretical constructs within a stated theory or at least one, but not all, of the stated predictors (see I5) are linked to at least one intervention technique	17(89)	0.4372	(-0.3287, 1.2031)	0.2632
Theory-relevant constructs are measured: post intervention (I12a)	At least one construct of theory (or predictor) mentioned in relation to the intervention is measured post intervention	18(95)	0.4372	(-0.3287, 1.2031)	0.2632
Theory-relevant constructs are measured: post and pre intervention (I12b)	At least one construct of theory (or predictor) mentioned in relation to the intervention is measured pre and post intervention	10(53)	0.0687	(-0.3625, 0.4999)	0.7548
Changes in measured theory-relevant constructs or predictors (I13)	The intervention leads to significant change in at least one theory relevant construct or predictor (vs control group) in favor of the intervention	9(47)	0.3521	(-0.0563, 0.7604)	0.0911
Mediator predicts the dependent variable (I14a)	Mediator predicts dependent variable, or change in mediator leads to change in dependent variable	2(11)	0.5009	(-0.0510, 1.0528)	0.0753
Mediator predicts dependent variable, controlling for the independent variable (I14b)	Mediator predicts dependent variable when controlling for independent variable	2(11)	0.5009	(-0.0510, 1.0528)	0.0753
Intervention does not predict the dependent variable when controlling the independent variable (I14c)	Intervention does not predict dependent variable when controlling for mediator	0(0)	/	/	/
Mediated effect is statistically significant (I14d)	Mediated effect is statistically significant	2(11)	0.5009	(-0.0510, 1.0528)	0.0753
Results discussed in relation to theory (I15)	Results are discussed in terms of the theoretical basis of the intervention	8(42)	0.0845	(-0.3097, 0.4787)	0.6745
Appropriate support for theory (I16)	Support for the theory is based on appropriate mediation, or refutation of the theory is based on obtaining appropriate null effects (i.e., changing behavior without changing the theory-relevant constructs)	2(11)	0.5009	(-0.0510, 1.0528)	0.0753
Results used to refine theory: adding or removing constructs to the theory (I17a)	Authors attempt to refine the theory upon which the intervention was based by adding or removing constructs to the theory	0(0)	/	/	/
Results used to refine theory: specifying that the interrelationships between the theoretical constructs should be changed(I17b)	Authors attempt to refine the theory upon which the intervention was based by specifying that the interrelationships between the theoretical constructs should be changed and spelling out which relationships should be changed	0(0)	/	/	/

**Theory coding scheme categories (category number)**	**Category description**	**Items included**	**univariate**		
			**B**	**95%CI**	**p**
Reference to underpinning theory (C1)	Stated or suggested, rather than demonstrated theoretical base	1, 2, 3	0.1113	(-0.0806, 0.3031)	0.2556
Targeting of relevant theoretical constructs (C2)	Whether evidence was provided that a targeted theoretical construct predicted behavior, whether theory or predictors were explicitly used for designing the intervention, and the extent to which the intervention targets particular theory-relevant constructs	2, 5, 6, 7, 8, 9, 10, 11	0.0540	(-0.0624, 0.1704)	0.3634
Using theory to select recipients or tailor interventions (C3)	Whether theory was used to select participants likely to benefit from the intervention, or to tailor the intervention to the needs of a particular individual	4, 6	0.0418	(-0.2365, 0.3201)	0.7685
Measurement of constructs (C4)	Whether the relevant theory-based constructs or predictors have been measured	12a, 12b	0.0751	(-0.1382, 0.2884)	0.4904
Testing of theory: mediation effects (C5)	Whether theoretical constructs are measured, whether the intervention changes the theoretical	12a, 12b, 13, 14a, 14b,	0.0660	(-0.0112, 0.1432)	0.0938

	constructs, and whether these changes explain the effect	14c, 14d, 15, 16			
Refining theory (C6)	Whether the results of evaluating theory-based interventions are used to refine theory	17a, 17b	/	/	/
Total use of theory	/	All items	0.0621	(0.0128, 0.1115)	0.0136*

* p-value < 0.05

Supporting Information 7： description of each item and category of the TCS

Theory coding scheme item (item number)	Item description
Theory or model of behavior mentioned(I1)	Models or theories that specify relations among variables to explain or predict behavior are mentioned even if the intervention is not based on this theory
Targeted construct mentioned as predictor of behavior (I2)	“Targeted” construct refers to a psychological construct that the study intervention is hypothesized to change
Intervention based on single theory (I3)	The intervention is based on a single theory (rather than a combination of theories or theory and predictors)
Theory or predictors used to select recipients for the intervention (I4)	Participants were screened or selected based on achieving a particular score or level on a theory-relevant construct or predictor
Theory or predictors used to select or develop intervention techniques (I5)	The intervention is explicitly based on a theory or predictor or combination of theories and predictors
Theory or predictors used to tailor intervention techniques to recipients (I6)	The intervention differs for different subgroups that vary on a psychological construct or predictor at baseline
All intervention techniques are explicitly linked to at least one theory-relevant construct or predictor (I7)	Each intervention technique is explicitly linked to at least one theory-relevant construct or predictor
At least one, but not all, of the intervention techniques are explicitly linked to at least one theory-relevant construct or predictor (I8)	At least one, but not all, of the intervention techniques are explicitly linked to at least one theory-relevant construct or predictor
Group of techniques are linked to a group of constructs or predictors (I9)	A cluster of techniques is linked to a cluster of constructs or predictors
All theory-relevant constructs or predictors are explicitly linked to at least one intervention technique (I10)	Every theoretical construct within a state theory, or every stated predictor, is linked to at least one intervention technique
At least one, but not all, of the theory-relevant constructs or predictors are explicitly linked to at least one intervention technique (I11)	At least one, but not all, of the theoretical constructs within a stated theory or at least one, but not all, of the stated predictors (see I5) are linked to at least one intervention technique
Theory-relevant constructs are measured: post intervention (I12a)	At least one construct of theory (or predictor) mentioned in relation to the intervention is measured post intervention
Theory-relevant constructs are measured: post and pre intervention (I12b)	At least one construct of theory (or predictor) mentioned in relation to the intervention is measured pre and post intervention
Changes in measured theory-relevant constructs or predictors (I13)	The intervention leads to significant change in at least one theory relevant construct or predictor (vs control group) in favor of the intervention
Mediator predicts the dependent variable (I14a)	Mediator predicts dependent variable, or change in mediator leads to change in dependent variable
Mediator predicts dependent variable, controlling for the independent variable (I14b)	Mediator predicts dependent variable when controlling for independent variable
Intervention does not predict the dependent variable when controlling the independent variable (I14c)	Intervention does not predict dependent variable when controlling for mediator
Mediated effect is statistically significant (I14d)	Mediated effect is statistically significant
Results discussed in relation to theory (I15)	Results are discussed in terms of the theoretical basis of the intervention
Appropriate support for theory (I16)	Support for the theory is based on appropriate mediation, or refutation of the theory is based on obtaining appropriate null effects (i.e., changing behavior without changing the theory-relevant constructs)
Results used to refine theory: adding or removing constructs to the	Authors attempt to refine the theory upon which the intervention was based by adding or removing constructs to the

theory (I17a)	theory
Results used to refine theory: specifying that the interrelationships between the theoretical constructs should be changed(I17b)	Authors attempt to refine the theory upon which the intervention was based by specifying that the interrelationships between the theoretical constructs should be changed and spelling out which relationships should be changed

**Theory coding scheme categories (category number)**	**Category description**
Reference to underpinning theory (C1)	Stated or suggested, rather than demonstrated theoretical base
Targeting of relevant theoretical constructs (C2)	Whether evidence was provided that a targeted theoretical construct predicted behavior, whether theory or predictors were explicitly used for designing the intervention, and the extent to which the intervention targets particular theory-relevant constructs
Using theory to select recipients or tailor interventions (C3)	Whether theory was used to select participants likely to benefit from the intervention, or to tailor the intervention to the needs of a particular individual
Measurement of constructs (C4)	Whether the relevant theory-based constructs or predictors have been measured
Testing of theory: mediation effects (C5)	Whether theoretical constructs are measured, whether the intervention changes the theoretical constructs, and whether these changes explain the effect
Refining theory (C6)	Whether the results of evaluating theory-based interventions are used to refine theory
Total use of theory	/

Supporting Information 8

Supporting Information 8.1 forest plot of lost to follow-up at 3-month (subgroups by intervention type)

Study or Subgroup	Experimental Events	Experimental Total	Control Events	Control Total	Weight	Risk Ratio MH, Random, 95% CI
subl = text–based						
Abroms–2014	55	262	31	241	5.6%	1.63 [1.09, 2.44]
Mavrot–2017	290	559	251	561	9.1%	1.16 [1.03, 1.31]
Rodgers–2005	106	852	65	853	7.0%	1.63 [1.22, 2.19]
Liao–2018	99	674	35	411	6.0%	1.72 [1.20, 2.49]
Nguyen–2019	639	1242	720	1236	9.5%	0.88 [0.82, 0.95]
Bricker–2020	171	1214	151	1201	8.2%	1.12 [0.91, 1.37]
Mussennei–2016	44	827	44	763	5.5%	0.92 [0.61, 1.38]
Michele–2012	30	76	24	75	5.3%	1.23 [0.80, 1.90]
Total (95% CI)		5706		5341	56.2%	1.22 [1.01, 1.48]
Heterogeneity: $Tau^2 = 0.055$; $Chi^2 = 46.72$, df = 7 ($P < 0.01$); $I^2 = 85\%$						
Test for overall effect: Z = 2.03 (P = 0.04)						
subl = multi–platform–based						
Baskerville–2018	394	820	354	779	9.3%	1.06 [0.95, 1.17]
BinDhim–2018	30	342	38	342	5.0%	0.79 [0.50, 1.24]
Brendryen–2008	10	144	24	146	3.0%	0.42 [0.21, 0.85]
Kraft–2008	15	197	20	199	3.4%	0.76 [0.40, 1.44]
Goldenhersch–2020	4	60	48	60	1.9%	0.08 [0.03, 0.22]
Scholten–2019	0	72	5	72	0.3%	0.09 [0.01, 1.61]
Whittaker–2011	31	110	29	116	5.2%	1.13 [0.73, 1.74]
Swartz–2006	84	171	70	180	7.7%	1.26 [1.00, 1.60]
Mays–2021	13	113	4	119	1.5%	3.42 [1.15, 10.19]
Garcia–Pazo–2021	11	61	14	49	3.0%	0.63 [0.32, 1.26]
Chulasai–2022	18	137	17	136	3.5%	1.05 [0.57, 1.95]
Total (95% CI)		2227		2198	43.8%	0.80 [0.59, 1.09]
Heterogeneity: $Tau^2 = 0.165$; $Chi^2 = 50.21$, df = 10 ($P < 0.01$); $I^2 = 80\%$						
Test for overall effect: Z = −1.40 (P = 0.16)						
**Total (95% CI)**		**7933**		**7539**	**100.0%**	**1.05 [0.91, 1.21]**
Heterogeneity: $Tau^2 = 0.057$; $Chi^2 = 92.11$, df = 18 ($P < 0.01$); $I^2 = 80\%$						
Test for overall effect: Z = 0.64 (P = 0.52)						
Test for subgroup differences: $Chi^2 = 5.10$, df = 1 (P = 0.02)						

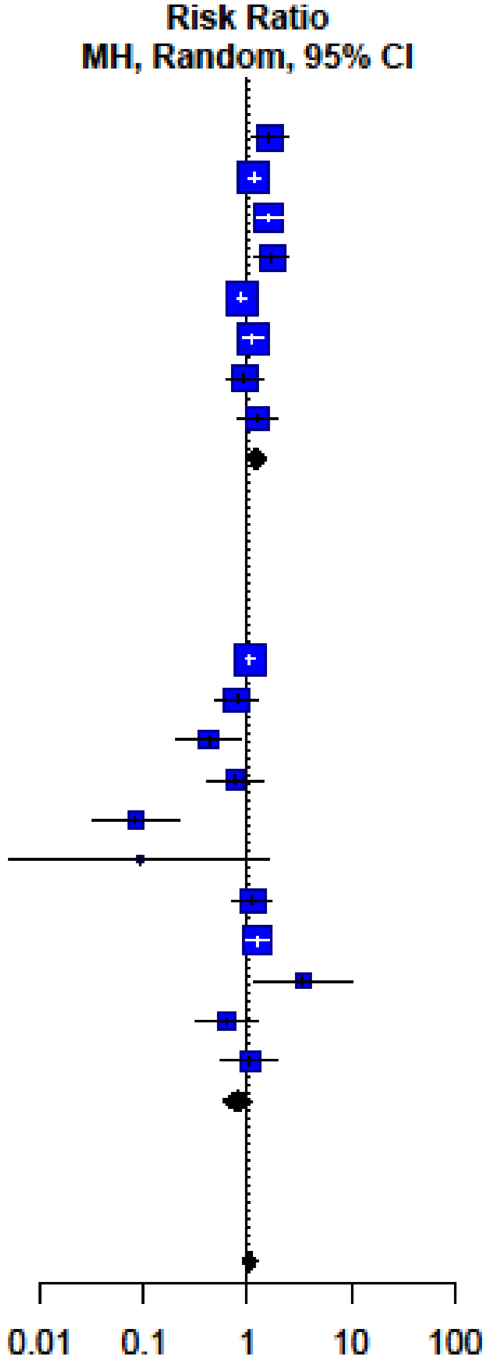

Supporting Information 8.2 forest plot of lost to follow-up at endpoint (subgroups by intervention type)

Study or Subgroup	Experimental Events	Experimental Total	Control Events	Control Total	Weight	Risk Ratio MH, Random, 95% CI
intervention = text–based						
Abroms–2014	52	262	70	241	6.0%	0.68 [0.50, 0.93]
Mavrot–2017	353	559	251	561	6.9%	1.41 [1.26, 1.58]
Rodgers–2005	261	852	179	853	6.7%	1.46 [1.24, 1.72]
Liao–2018	113	674	35	411	5.8%	1.97 [1.38, 2.82]
Nguyen–2019	732	1242	793	1236	7.0%	0.92 [0.86, 0.98]
Bricker–2020	174	1214	134	1201	6.5%	1.28 [1.04, 1.59]
Mussennei–2016	44	827	44	763	5.5%	0.92 [0.61, 1.38]
Michele–2012	30	76	24	75	5.4%	1.23 [0.80, 1.90]
Total (95% CI)		5706		5341	49.8%	1.18 [0.95, 1.47]
Heterogeneity: $Tau^2 = 0.083$; $Chi^2 = 83.02$, df = 7 ($P < 0.01$); $I^2 = 92\%$						
Test for overall effect: Z = 1.48 (P = 0.14)						
intervention = multi–platform–based						
Baskerville–2018	394	820	354	779	6.9%	1.06 [0.95, 1.17]
BinDhim–2018	48	342	53	342	5.8%	0.91 [0.63, 1.30]
Brendryen–2008	26	144	38	146	5.3%	0.69 [0.45, 1.08]
Kraft–2008	24	197	31	199	5.0%	0.78 [0.48, 1.28]
Goldenhersch–2020	4	60	48	60	2.8%	0.08 [0.03, 0.22]
Scholten–2019	0	72	5	72	0.5%	0.09 [0.01, 1.61]
Whittaker–2011	35	110	26	116	5.4%	1.42 [0.92, 2.19]
Swartz–2006	84	171	70	180	6.4%	1.26 [1.00, 1.60]
Mays–2021	15	113	6	119	3.0%	2.63 [1.06, 6.55]
Garcia–Pazo–2021	23	61	15	49	4.8%	1.23 [0.72, 2.09]
Chulasai–2022	18	137	17	136	4.3%	1.05 [0.57, 1.95]
Total (95% CI)		2227		2198	50.2%	0.87 [0.56, 1.36]
Heterogeneity: $Tau^2 = 0.462$; $Chi^2 = 43.61$, df = 10 ($P < 0.01$); $I^2 = 77\%$						
Test for overall effect: Z = −0.61 (P = 0.54)						
**Total (95% CI)**		**7933**		**7539**	**100.0%**	**1.04 [0.85, 1.28]**
Heterogeneity: $Tau^2 = 0.160$; $Chi^2 = 126.76$, df = 18 ($P < 0.01$); $I^2 = 86\%$						
Test for overall effect: Z = 0.40 (P = 0.69)						
Test for subgroup differences: $Chi^2 = 1.44$, df = 1 (P = 0.23)						

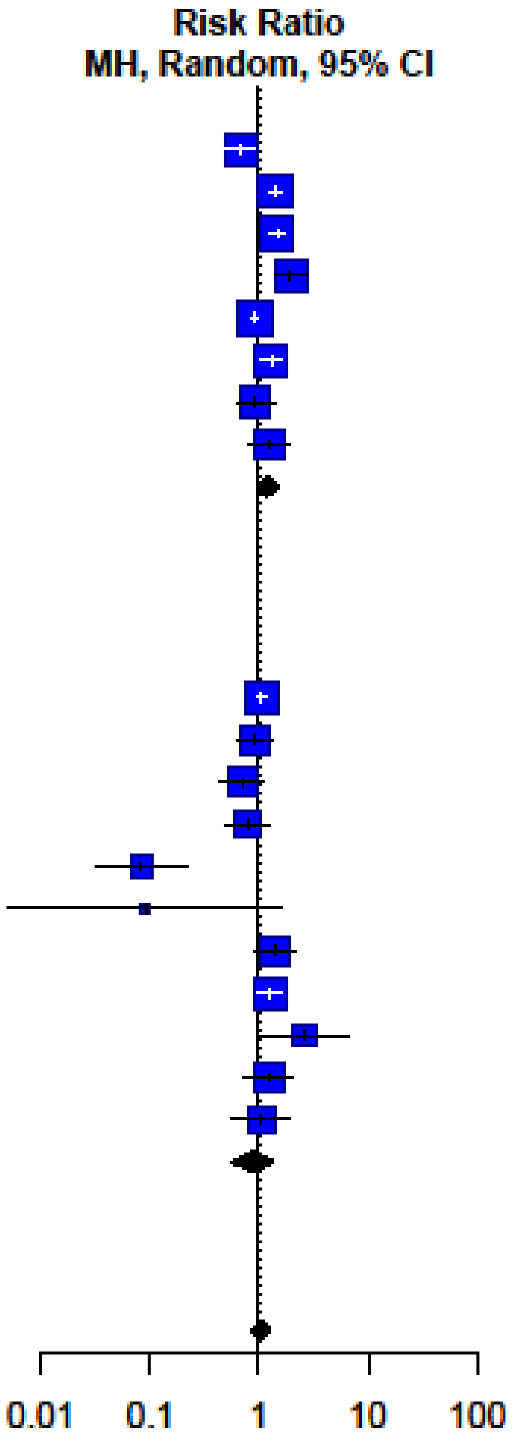

Supplement: Multimedia Appendix 1 [file jmir_v24i11e38206_app1.pdf]
